# Mutational signature assignment heterogeneity is widespread and can be addressed by ensemble approaches

**DOI:** 10.1093/bib/bbad331

**Published:** 2023-09-22

**Authors:** Andy J Wu, Akila Perera, Linganesan Kularatnarajah, Anna Korsakova, Jason J Pitt

**Affiliations:** Cancer Science Institute of Singapore, National University of Singapore, Singapore, Singapore; School of Medicine, National University of Singapore, Singapore, Singapore; Cancer Science Institute of Singapore, National University of Singapore, Singapore, Singapore; School of Computing, National University of Singapore, Singapore, Singapore; Cancer Science Institute of Singapore, National University of Singapore, Singapore, Singapore; Cancer Science Institute of Singapore, National University of Singapore, Singapore, Singapore; Cancer Science Institute of Singapore, National University of Singapore, Singapore, Singapore; NUS Centre for Cancer Research, Yong Loo Lin School of Medicine, National University of Singapore, Singapore, Singapore; Genome Institute of Singapore, Agency for Science, Technology and Research (A*STAR), Singapore, Singapore

**Keywords:** cancer, genomics, mutational signatures, ensemble, benchmark, web portal

## Abstract

Single-base substitution (SBS) mutational signatures have become standard practice in cancer genomics. In lieu of *de novo* signature extraction, reference signature assignment allows users to estimate the activities of pre-established SBS signatures within individual malignancies. Several tools have been developed for this purpose, each with differing methodologies. However, due to a lack of standardization, there may be inter-tool variability in signature assignment. We deeply characterized three assignment strategies and five SBS signature assignment tools. We observed that assignment strategy choice can significantly influence results and interpretations. Despite varying recommendations by tools, Refit performed best by reducing overfitting and maximizing reconstruction of the original mutational spectra. Even after uniform application of Refit, tools varied remarkably in signature assignments both qualitatively (Jaccard index = 0.38–0.83) and quantitatively (Kendall tau-b = 0.18–0.76). This phenomenon was exacerbated for ‘flat’ signatures such as the homologous recombination deficiency signature SBS3. An ensemble approach (EnsembleFit), which leverages output from all five tools, increased SBS3 assignment accuracy in *BRCA1/2*-deficient breast carcinomas. After generating synthetic mutational profiles for thousands of pan-cancer tumors, EnsembleFit reduced signature activity assignment error 15.9–24.7% on average using Catalogue of Somatic Mutations In Cancer and non-standard reference signature sets. We have also released the EnsembleFit web portal (https://www.ensemblefit.pittlabgenomics.com) for users to generate or download ensemble-based SBS signature assignments using any strategy and combination of tools. Overall, we show that signature assignment heterogeneity across tools and strategies is non-negligible and propose a viable, ensemble solution.

## INTRODUCTION

Mutational signatures are genome-wide patterns of somatic mutations accumulated throughout the lineage of a cell. These fixed patterns are scars of semi-random mutations generated via defective DNA repair processes or increased DNA damage—from both endogenous and exogenous sources. As such, mutational signatures serve as a high-level assessment of genome instability, which is a hallmark of cancer. With reduced costs of next-generation sequencing, the study of mutational signatures in cancer has been increasing over the past decade. By extracting signatures from thousands of pan-cancer tumor samples, the Pan-cancer Analysis of Whole Genomes (PCAWG) Working Group 7 has identified over 100 signatures that are currently stored in the Catalogue of Somatic Mutations In Cancer (COSMIC) database [1]. The etiology of signatures ranges from specific mutators such as APOBEC activity [2] to cellular phenotypes such as homologous recombination deficiency (HRD) [3, 4] and even broader associations like aging [1] or tobacco smoking [5]. Hence, identifying the presence and activities (i.e. exposures) of mutational signatures in malignancies can provide clues to cancer pathophysiology or discover therapeutic targets.

Many mutational signature analysis tools have been built over the years with the aim to extract novel signatures and/or assign known signatures using somatic mutations of tumors [6–8]. Generally, most tools employ a dimensionality reduction method known as non-negative matrix factorization (NMF) [9]. This approach estimates signatures, each in the form of matrices, by factorizing a mutational catalog of observed counts of mutation types in a given set of samples. The most well-studied mutation type is the single-base substitution (SBS), which features six strand-agnostic pyrimidine base substitutions (C > A, C > G, C > T, T > A, T > C and T > G) under all possible combinations of bases adjacent (1 bp up- and downstream) to the substitution. This is canonically known as the trinucleotide context and forms the 96 channels (SBS96) that constitute a signature’s profile. Even though *de novo* signature extraction is a popular method in studies of SBS mutational signatures, it has some disadvantages. Extraction is computationally intensive as it aims to use patients’ mutational profiles to optimize for two unknown matrices: a signature set defined by SBS96 channels and the activities of those signatures within each patient. This can require significant computational resources to optimize these matrices for large sample cohorts (i.e. *n* > 1000). The number of signatures extracted also depends on the diversity of the dataset; extraction on a set of samples without sufficiently differential mutational profiles could result in one or few composite signatures [1]. *De novo* signatures are conventionally compared back to an existing set of reference signatures (e.g. COSMIC) using a distance metric, typically cosine similarity, with arbitrary cutoffs for calling identities [8, 10]. In contrast, reference assignment only estimates the activities of existing signatures, such as those from COSMIC, which is less computationally intensive and agnostic to the diversity of the input dataset. The use of a reference signature set also allows for the standardization of signature definitions across studies, which removes a point of variability and subsequent user friction that exists in *de novo* extraction. Hence, in the absence of identifying or expecting to identify novel signatures, reference assignment is a far more accessible and practical approach for mutational signature analyses.

Despite the benefits of employing reference assignment for mutational signature analysis, the current lack of standardization can disrupt repeatability and biological interpretations. One major challenge is overfitting in which samples are assigned too many signatures including ones that were not expected to be active in the cancer type [11]. This issue is a side effect of optimizing matrix reconstructions with minimal to no constraints on the number of signatures. To mitigate overfitting, mutational signature tool developers have proposed multiple assignment strategies including pre-processing or *ad hoc* filtering of the reference signature set and fine-tuning of tool parameters [11]. Aside from potentially disparate decisions on reference set filtering, the underlying optimization method for the matrix deconvolution often varies by tool. The choice of optimizer can depend on the distance metric used in assessing the matrix reconstruction (e.g. Euclidean distance and Kullback–Leibler divergence) and the assumption of the convexity of the solution [9, 12, 13]. Some common optimization methods include non-negative least squares, quadratic programming and simulated annealing [14–17]. Some evidence suggests that optimizers can perform significantly different assignments depending on the sample [8, 18]. Additionally, high intra-tool assignment variability has been observed for certain signatures [18]. It was previously shown that the employment of different mutational signature analysis tools—which are a culmination of heuristics, strategies and computational engines—can affect the agreement of *de novo* extraction and assignment [10, 18]. Nonetheless, the extent of this variation across multiple tools and strategies, and how it can influence biological interpretations, is unknown. There is a growing need for comprehensive studies to evaluate and provide recommendations for SBS mutational signature assignment.

**Figure 1:**
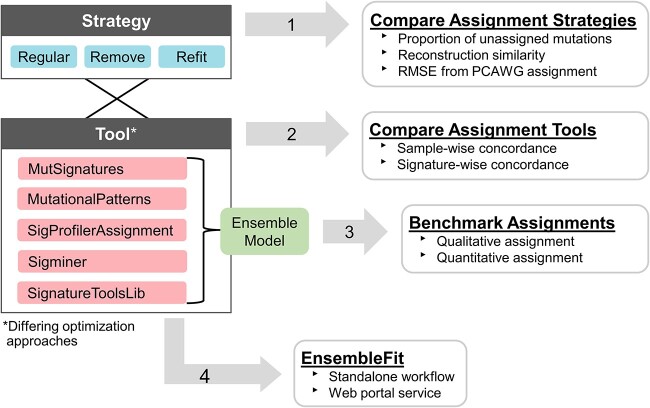
Overview of the four main study objectives. After implementing the assignment strategies for all five tools, (i) assignment strategies on the PCAWG dataset (*n* = 2780) were compared on three metrics: the proportion of mutations that were unassigned; the reconstruction similarity of the assignment; and the root mean squared error (RMSE) of the assignment from PCAWG’s published assignment. (ii) Using the same strategy, the tools were compared to assess sample-wise and signature-wise concordance. (iii) Ensemble-based model that integrates the assignments of the five tools was developed and benchmarked. (iv) The EnsembleFit workflow was made available as a standalone package or a web portal service. Additional algorithmic details for the assignment tools and strategies can be found in Table 1 and Supplementary Methods.

Here, we assessed the consistency of mutational signature assignments across five state-of-the-art tools—MutSignatures [14], MutationalPatterns [15], SigProfilerAssignment, Sigminer [16] and SignatureToolsLib [17]—on real and simulated SBS mutational catalogs. We first implemented three common assignment strategies both to evaluate their merits and allow fairer comparison across tools. Then, we measured inter-tool assignment concordance across cohorts, samples and individual signatures—with particular emphasis on challenging signatures such as SBS3. Information was leveraged from all tools to create an integrative model for qualitative and quantitative signature assignments. We demonstrate that this ensemble approach improves the accuracy of SBS3 assignment, a marker of homologous recombination deficiency (HRD), using a large collection of breast cancer tumors with known *BRCA1/2* status. Synthetic mutational profiles were used to show this approach consistently outperformed individual tools regardless of cancer type or reference signature catalog. The entire workflow has also been packaged as a web portal, named EnsembleFit, for users to easily generate and assess ensemble signature assignments across tools and assignment strategies. A summary of these study objectives can be found in Figure 1.

## MATERIALS AND METHODS

### Mutational catalogs

The PCAWG7 SBS96 mutational catalog, retrieved from https://dcc.icgc.org/releases/PCAWG/mutational_signatures/Input_Data_PCAWG7_23K_Spectra_DB/Mutation_Catalogs_--_Spectra_of_Individual_Tumours, features 2780 pan-cancer tumors (37 cancer types)—including 198 breast adenocarcinoma (BRCA) tumors. The 560 breast cancer (BRCA-EU) SBS catalog was generated using SigProfilerMatrixGenerator and variant call format (VCF) files generated by parsing simple somatic mutation files downloaded from https://dcc.icgc.org/api/v1/download?fn=/current/Projects/BRCA-EU/simple_somatic_mutation.open.BRCA-EU.tsv.gz. No somatic mutation filtering was performed.

### Mutational signature assignment tools

Reference signature assignment aims to solve the matrix deconvolution problem V ≈ W × H where V, the mutational catalog of observed somatic mutations in the samples, and W, the reference signature set, are known—while H, the activities of the signatures in the samples, is to be estimated. We chose the established tools SigProfilerAssignment 0.0.13 [10] and SignatureToolsLib 2.1.2 [17] due to their usage by pioneering consortia such as the International Cancer Genome Consortium's (ICGC) PCAWG [1] and Genomics England [19]. We complemented these with three additional tools: MutSignatures 2.1.1 [14], MutationalPatterns 3.4.1 [15] and Sigminer 2.1.7 [16]. These tools were selected as they were recently published with demonstrated functional or performance improvements over existing tools and have been subsequently used in independent studies [20–25]. The tools largely differ in the optimization algorithm used to estimate H (Table 1) as well as the metrics and thresholds used during unsupervised learning. Assignment strategy implementation details for all tools can be found in Supplementary Methods.

**Table 1:** Overview of mutational signature assignment tools with their assignment strategies and recommendations.

Tool name	Default strategy	Recommended strategy	Optimizer	Implementation of strategy		
				Regular	Remove	Refit
MutSignatures	Regular	Refit	FC-NNLS	0% Threshold	<5% → Unassigned	Subset signatures (≥5%) for Regular
MutationalPatterns	Regular/Refit	Refit	NNLS	0% Threshold	<5% → Unassigned	Tool’s implementation
SigProfilerAssignment	Refit	Refit	NNLS	0.01% Threshold	<5% → Unassigned	Tool’s implementation
Sigminer	Regular	Remove	QP	0% Threshold	<5% → Unassigned	Subset signatures (≥5%) for Regular
SignatureToolsLib	Remove	Remove	MU	0% Threshold	<5% → Unassigned	Subset signatures (≥5%) for Regular

Note: (1) The Refit implementation in this study required SBS1 and SBS5 to be included in the subset signature reference (see Supplementary Methods). (2) Details of the individual tool's algorithm can also be found in Supplementary Methods. (3) FC-NNLS: Fast Combinatorial Non-Negative Least Square. NNLS: Non-Negative Least Squares. QP: Quadratic Programming. MU: Multiplicative Update Rule.

### Sample-wise concordance of signature assignments

The PCAWG and PCAWG-BRCA datasets were first filtered for valid samples; samples in which correlation can be calculated for all 10 pairwise comparisons of five tools. A sample was considered invalid if it was assigned fewer than two signatures by any tool. Out of 2780 PCAWG samples, 17 samples were deemed invalid. Signature activity correlations (Kendall tau-b) were determined for each valid sample and pair of tools. Signatures assigned an activity level of 0 by both tools were not included in this calculation. For a given pair of tools, the reported sample-wise correlation is the mean Kendall tau-b for all valid samples within the cohort. To score the agreement of two tools, the Jaccard index (J) was calculated using


$$ J\left(A,B\right)=\frac{\left|A\cap B\right|}{\left|A\cup B\right|} $$

where *A* and *B* are sets of signatures assigned by tool A and tool B, respectively. The overall sample-wise agreement of the two tools is the mean of all valid samples’ Jaccard indices.

### Signature-wise concordance of signature assignments

The signature reference set is first filtered for valid signatures: signatures in which correlation can be calculated for all 10 pairwise comparisons of five tools. For every pair, a signature is invalid if there are no samples with >0 activity assigned by any tool of that pair, or if the number of samples with at least one tool assigning >0 activity is less than two. Kendall tau-b was then used to correlate a signature’s assigned activity between two tools. If a signature was deemed inactive by both tools, those samples were excluded from this calculation. The assignment correlation of a valid signature is the mean Kendall tau-b for all pairwise combinations of tools.

### Integration of tools’ assignments using an ensemble approach

If a signature has been assigned (activity >0) in a sample by three or more tools (i.e. the majority), it is considered assigned by the Ensemble-Majority model. If the assignment is done by all five tools, it is considered assigned by the Ensemble-Unanimous model. The quantitative integration, the Ensemble-Mean model, is done using a per-signature bootstrap resampling of means (*n* = 500) across the five tools to estimate the mean activity value (see Supplementary Methods for details). The estimated activities of all signatures of that sample are then standardized to ensure that they sum to 1.

### Benchmarking qualitative assignment of SBS3 in *BRCA1/2*-deficient samples

The true-positive (TP), false-positive (FP), true-negative (TN) and false-negative (FN) values were calculated for each tool’s assignment of SBS3 across 560 BRCA-EU patients. True positive is when a tool assigns SBS3 to a *BRCA1/2* bi-allelic loss sample (*n* = 77) while a TN is when SBS3 is not assigned in *BRCA1/2*-proficient samples (*n* = 483). The positive predictive value (PPV), negative predictive value (NPV) and diagnostic accuracy were calculated based on the following formulas:


$$ \mathrm{PPV}=\frac{\mathrm{TP}}{\mathrm{TP}+\mathrm{FP}} $$

$$ \mathrm{NPV}=\frac{\mathrm{TN}}{\mathrm{TN}+\mathrm{FN}} $$

$$ \mathrm{Accuracy}=\frac{\mathrm{TP}+\mathrm{TN}}{\mathrm{TP}+\mathrm{FP}+\mathrm{TN}+\mathrm{FN}} $$

### Benchmarking quantitative assignment

Synthetic datasets were generated based on a previously described method using SynSigGen [1, 10] which simulates SBS patterns in tumors based on real tumors’ signature activities. The published SigProfiler and SignatureAnalyzer signature activities of the PCAWG dataset were used to generate the respective synthetic datasets following the same dataset heterogeneity and signature activity distributions. The method outputs a synthetic signature activities matrix and a synthetic SBS96 mutational catalog. The mutational catalog and the corresponding reference signature matrix are used by the signature assignment tools to estimate the signature activities matrix, which is compared against the known synthetic signature activities. When comparing a tool’s estimated signature activities with the known activities, only the active synthetic signatures were considered when calculating the assignment error via the root mean squared error (RMSE). For each synthetic cohort, one RMSE was calculated for each tool using all samples.

## RESULTS AND DISCUSSIONS

### Implementing signature assignment strategies

**Figure 2:**
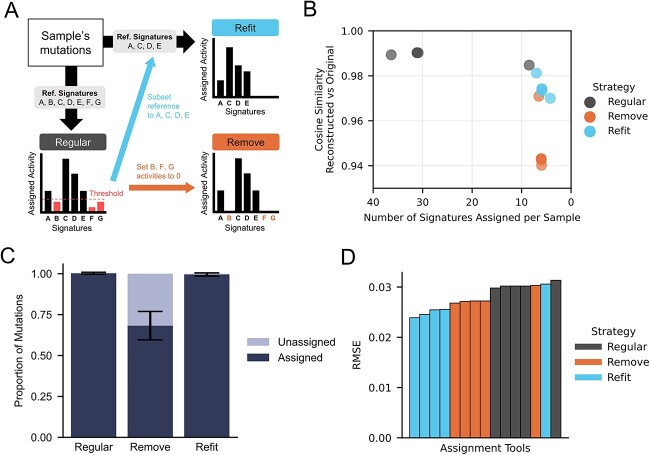
Comparison of signature assignment strategies using PCAWG (*n* = 2780). (**A**) Schematic diagram of the signature assignment strategies: Regular; Remove; and Refit. The Regular strategy assigns all signatures [named Reference (Ref.) Signatures A to G in the diagram]. Remove strategy removes assignments of Ref. Signatures B, F and G that have activity levels less than a set threshold. This is done by setting their activities to 0. Refit strategy subsets the reference signatures to keep only signatures above the threshold (Ref. Signatures A, C, D and E) and re-runs the assignment. (**B**) The relationship between two assignment performance metrics across the three strategies: the similarity between the reconstructed and original mutational spectrums (RvO similarity); and the number of signatures assigned per sample. The mean value of these two metrics is plotted for each tool-strategy combination. Darker circles indicate overlapping data points. (**C**) The proportion of mutations attributed to a signature for each assignment strategy. Proportion data are aggregated across all pairwise combinations of samples and tools (*n* = 5) with mean and standard deviation depicted. (**D**) Assessment of tool-strategy signature assignment accuracy (RSME) using the observed activity published by the PCAWG consortium [1] as a reference.

Given an existing set of reference signatures, sample-wise signature assignment canonically employs one of three strategies: ‘Regular’, ‘Remove’ and ‘Refit’ (Figure 2A). The Regular strategy performs assignment without a priori modification of the reference signature set, allowing all signatures an opportunity to be assigned to the sample. However, this approach may be prone to over-assigning signatures, particularly those at low activity levels [14, 17]. The Remove strategy has been proposed to mitigate this effect by setting a minimum threshold, typically 5%, for a signature to be considered active. Mutations assigned to signatures beneath this threshold will be considered unassigned (i.e. removed). While this approach may prevent signature overfitting, it may fail to utilize all mutations found within a sample. This could be particularly problematic when the total number of mutations is small (e.g. targeted capture sequencing). Despite this, the number of mutations discarded by Remove has not been thoroughly investigated. The Refit strategy has been proposed to simultaneously minimize overfitting while maximizing the number of assigned mutations (i.e. mutation utilization). Refit first identifies a subset of reference signatures with assigned activities equal to or greater than a desired threshold. Then, the Regular strategy is applied while only using the subset of signatures already determined to be active. Further differences between these strategies are provided in Supplementary Methods.

We investigated five commonly used mutational signature assignment tools and summarized their supported assignment strategies, recommendations and algorithmic differences in Table 1 and Supplementary Methods. All tools implement the Regular strategy by default since it is the core assignment method. While each tool recommends either Remove or Refit, these strategies were not always natively implemented. Although Sigminer implements the approach of removing mutations based on a set threshold, the mutations were not reassigned to an ‘Unassigned’ category, hence it was re-implemented in this study. A variation of Refit, one that iteratively subsets the reference, has been natively implemented by SigProfilerAssignment and MutationalPatterns. Overall, we extended MutSignatures, MutationalPatterns and SigProfilerAssignment to support Remove as well as MutSignatures, Sigminer and SignatureToolsLib to support Refit (see Supplementary Methods). This approach enabled us to assess which assignment strategy consistently performed the best across tools.

### Evaluating signature assignment strategies

Our first objective was to compare the general performance of assignment strategies irrespective of the tool being used. Using the whole genome sequencing (WGS)-derived SBS profiles representing 2780 individuals from the PCAWG project, we assigned COSMIC v3 SBS96 signatures (*n* = 78) to each sample using all combinations of tools and assignment strategies. For each sample and assignment, we extracted three performance metrics: the number of reference signatures assigned; the proportion of mutations assigned to signatures; and the cosine similarity of the reconstructed SBS96 mutational spectra versus the original (RvO). Generally, better assignment is achieved when the former metric is minimized to avoid overfitting and as the latter two each approach 1.

While the Regular strategy best reconstructs the original SBS mutational spectra (Figure 2B), most tools under this strategy assigned over 30 signatures per sample on average with the majority having low (<5%) activity levels. This may be an indication of overfitting in circumstances where a large number of signatures—regardless of known etiologies—were leveraged to reconstruct a profile as close to the original as possible. Consistent with this assertion, only a minority (~30%) of the signatures assigned by Regular are known to be active within each sample’s respective cancer type (Supplementary Figure 1; see Materials and Methods). For example, we found that 67 samples across 15 cancer types, including those affecting the central nervous system and visceral organs, were assigned SBS7c (ultraviolet light exposure) by the majority of the five tools. This signature is almost exclusively active in sun-exposed tissues such as skin melanoma. In contrast, both Remove and Refit rendered many fewer signatures active (Figure 2B) and those signatures were substantially more reflective of reported cancer type–specific activities (50–53% and 55–85%, respectively) compared to Regular (26– 52%) (Supplementary Figure 1). Refit achieved this outcome with a minimal drop in RvO, but Remove resulted in SBS sets that were less reflective of the original mutational spectra (Figure 2B). While Regular and Refit strategies assigned nearly all SBS (~99.5–100%) to a reference signature, Remove failed to assign nearly one-third (~32%) of the mutations in a given sample on average (Figure 2C). We postulated that Remove’s poorer RvOs may be due to non-random mutation removal and that this effect worsens as the proportion of mutations removed increases. Indeed, we observed non-random removal across SBS96 channels (Supplementary Figure 2) as well as a strong negative correlation between RvO and the proportion of unassigned mutations (τ_b_ = −0.54 – −0.36, *P* < 0.0001) (Supplementary Figure 3). Since cosine similarity is invariant to scaling but not to shifts [26], the weaker RvOs are not due to fewer assigned mutations, but rather, to shifting mutational spectra. Although Remove does mitigate overfitting, the non-random loss of mutations distorts the overall mutational spectra and could confound biological conclusions stemming from downstream analyses.

As a complement to the aforementioned metrics, we also compared the output of assignment strategies to published SBS96 exposures by PCAWG. The RMSE was calculated across all samples (see Materials and Methods). Generally, Refit performs the best, followed by Remove then Regular. Only SigProfilerAssignment under Regular and Remove and MutationalPatterns under Refit deviated from this pattern (Figure 2D, Supplementary Table 2). All tools except one, MutationalPatterns, perform best under Refit (Figure 2D, Supplementary Table 2). Even for the three tools that do not natively support Refit, our implementation led to a notable reduction in assignment error over Regular or Remove. Taken together, our results demonstrate that the Refit strategy—regardless of each tool’s native implementations or recommendations—should be the preferred assignment strategy due to a reduction in overfitting, proper mutational spectra reconstruction (Figure 2B), minimal mutation loss (Figure 2C) and improved assignment accuracy with *de facto* standard datasets (Figure 2D). Throughout the remainder of this study, we utilized the Refit strategy, which provided a conservative estimate of possible assignment heterogeneity across tools.

### Sample-wise signature assignment variation across tools

**Figure 3:**
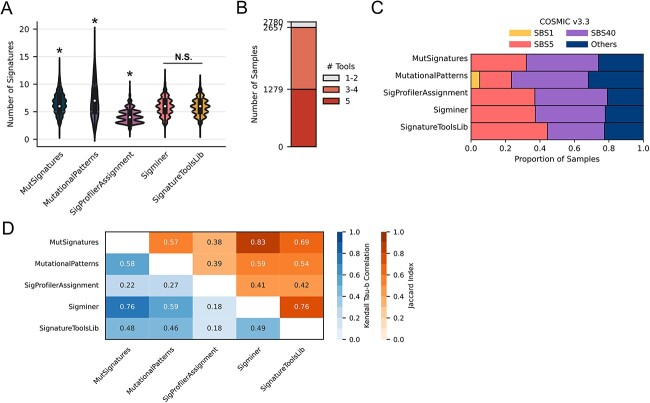
Sample-wise signature assignment agreement and correlation across tools with the Refit strategy. (**A**) Distribution of the number of signatures assigned to each PCAWG sample (*n* = 2780). Pairwise Kolmogorov–Smirnov tests were performed, and an asterisk denotes that the distribution of the tool is significantly different from every other tool. Sigminer and SignatureToolsLib (*P* = 0.88) are not significantly different (N.S.). (**B**) Consensus of assigned dominant signatures for each PCAWG sample. (**C**) Proportion of the assigned dominant signatures for each PCAWG sample; ‘others’ are signatures with ˂0.05 proportion (5%). (**D**) For each pair of tools and all samples, the mean correlation (Kendall tau-b) of signature activities (proportion) and the agreement (Jaccard index) of the set of active signatures.

We next assessed how the assignment of signature activity within PCAWG (*n* = 2780) varies by tool. Exploring the total number of signatures assigned per sample—MutSignatures, MutationalPatterns and SigProfilerAssignment differed from all other tools in both their mean (*P* < 0.05; Welch T-test) and distribution (*P* < 0.05; two-sample Kolmogorov–Smirnov test) (Figure 3A). SigProfilerAssignment assigned the fewest signatures with the lowest variance (mean = 4.26, SD = 1.27) while MutationalPatterns assigned the most signatures with the highest variance (mean = 7.01, SD = 3.18). Furthermore, not only did tools identify differing dominant signatures (i.e. ones with the highest activity) within samples (Figure 3B) but also altered which signature was most dominant across the entire cohort (Figure 3C).

To determine the overall agreement of signature assignments, we calculated pairwise overlap (Jaccard index) of assigned signatures and pairwise correlations (Kendall tau-b) of their activities for all tools and across each sample (see Materials and Methods). The Jaccard index (*J*) and mean correlation (τ_b_) for all samples were used to represent the qualitative agreement and quantitative correlation between any two tools, respectively (Figure 3D). Signatures deemed active by any two tools varied greatly as overlap ranged from partial (*J* = 0.45; MutationalPatterns with SigProfierAssignment) to high (*J* = 0.86; MutSignatures with Sigminer). Additionally, while all tools were correlated with one another—the strength of this correlation varied drastically (mean τ_b_ = 0.35–0.83). This pattern held when considering cancer types in isolation (Supplementary Figure 4). MutSignatures and Sigminer had the greatest agreement on assigned signatures (mean *J* = 0.86) as well as their estimated activities (mean τ_b_ = 0.83) despite running different optimizers (Table 1). This suggests that optimization methods alone are not always the major source of assignment disparities. Interestingly, despite having similar distributions of the total number of assigned signatures (Figure 3A), Sigminer and SignatureToolsLib were not the most concordant tools either qualitatively or quantitatively (Figure 3D, Supplementary Figure 5). Even though SigProfilerAssignment and MutationalPatterns have native implementations of the Refit strategy, their agreement and correlation were weakest (mean *J* = 0.45, mean τ_b_ = 0.35). Importantly, the tool-based heterogeneity observed within individual samples was often more substantial than the mean across the cohort (Supplementary Figure 6). This outcome is particularly problematic in the precision oncology setting where mutational signatures have been proposed as biomarkers for individualized therapy [27].

### Signature-wise signature assignment variation across tools

We then aimed to determine if cross-tool heterogeneity disproportionately affected certain signatures. Using 198 breast cancer samples from PCAWG, we correlated per signature activity assignments across all pairs of tools (see Materials and Methods). We also calculated the Shannon diversity index (SDI) for each signature profile as an estimate of ‘flatness’—that is, the uniformity of feature contributions across the SBS96 spectrum. Correlations varied widely across signatures ranging from perfect to weak including two signatures with negative correlations (mean τ_b_ = −0.26–1.0). This wide range is also held with pan-cancer samples (Supplementary Figure 7A). Notably, the mean correlation for each signature is inversely proportional to its SDI, and this relationship is clearly evident across all cancer types (Supplementary Figure 7B) (τ_b_ = −0.321, *P* = 0.0)—suggesting that flatter signatures have less consistency across tools. SBS17b, which has an unknown etiology, has a perfect correlation (τ_b_ = 1.0) across all tools. This is the only COSMIC v3.3 signature with prominent features in the T > G substitution class, which could help facilitate unambiguous assignment. Three highly-studied signatures of known etiology—SBS13 (APOBEC), SBS2 (APOBEC) and SBS1 (aging)—demonstrated consistent activity estimates across tools (mean τ_b_ = 0.92, 0.90 and 0.90, respectively). Among signatures with weak correlation (τ_b_ < 0.30), 44.4% (4 out of 9) have etiologies in DNA damage repair and 33.3% (3 out of 9) have etiologies in mutagen exposures (Supplementary Table 4). Inconsistent activity assignment of signatures with accepted etiologies may lead researchers to draw differing biological conclusions depending on the tool used.

**Figure 4:**
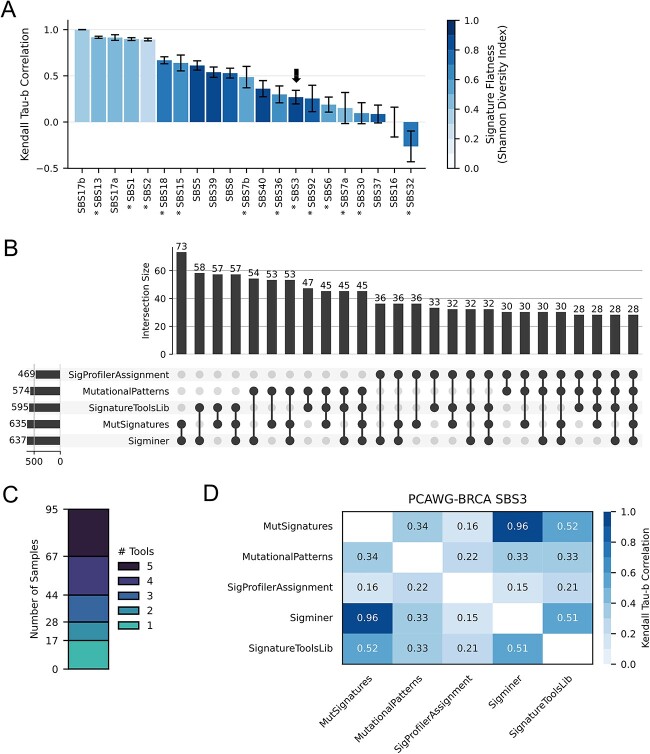
Assignment correlation of individual signatures by different tools using the Refit strategy. Across PCAWG-BRCA samples (*n* = 198), (**A**) signature-wise correlation (Kendall tau-b) of assigned frequencies across all five tools. Each signature’s profile flatness is represented by Shannon’s diversity index. An asterisk (*) prefixing a signature indicates a proposed etiology in COSMIC v3.3. The HRD-associated signature SBS3 has been denoted with an arrow. (**B**) Agreement of qualitative SBS3 assignment across different tools with an UpSet plot providing all possible intersections. (**C**) The number of samples assigned SBS3 by one or more tools is shown as a stacked bar chart. (**D**) Heat map depicting the correlation of quantitative SBS3 activity assignment across tools via mean per-sample correlation (Kendall tau-b) (see Materials and Methods).

SBS3—often observed in conjunction with *BRCA1/2* inactivation—has been proposed as a marker of HRD [27, 28]. Malignancies exhibiting HRD are eligible for platinum-based or PARP1 inhibition therapy [27]. Given the clinical implications of HRD-positivity, we assessed how the choice of assignment tool could affect SBS3-based HRD calling both qualitatively and quantitatively. Ninety-five of the 198 PCAWG-BRCA samples had SBS3 assigned by at least one tool (Figure 4B and C). Of these, only 28 (~29%) were unanimously assigned SBS3 by all five tools (Figure 4B). Depending on the tool used, the presumed HRD-positivity rate nearly doubled from 19.7% (SigProfilerAssignment) to 37.9% (Sigminer). Even when tools agreed that SBS3 is present, their quantitative activity estimates were often poorly correlated. For each pair of tools, Kendall tau-b correlation was calculated across all samples where both tools assigned SBS3 (Figure 4D). Sigminer and MutSignatures had the strongest correlation (τ_b_ = 0.96), while SigProfilerAssignment had the weakest correlation with all other tools (τ_b_ = 0.15–0.22). This result is in stark contrast to the APOBEC-associated signatures SBS2 and SBS13, which have strong pairwise correlations between tools (all τ_b_ > 0.9) (Supplementary Figure 8). Altogether, we observed that the discordance of a signature’s assignment is positively related to the flatness of its spectrum. This could disrupt the clinical utility of signatures with potentially actionable etiologies (i.e. SBS3).

### Qualitative and quantitative benchmarking of ensemble signature assignment

**Figure 5:**
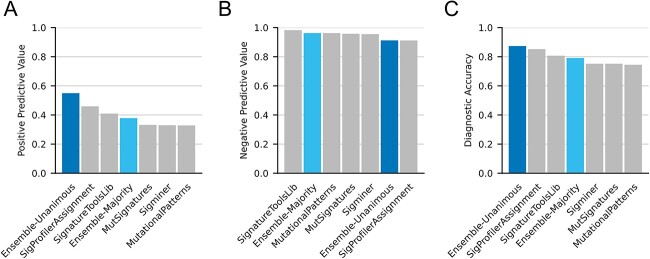
Benchmarking SBS3 assignment using BRCA1/2-deficient and -proficient breast tumors. Assessing for peer review the presence of SBS3 in whole genome-sequenced tumors from 560 breast cancer patients (BRCA-EU) with confirmed BRCA1/2 bi-allelic loss status (*n* = 77). The PPV, NPV and accuracy of SBS3 as a surrogate for BRCA1/2 bi-allelic loss (i.e. BRCA-deficiency) are provided for all approaches.

We hypothesized that an ensemble approach that leverages all tools simultaneously could improve signature- and sample-wise assignments, both qualitatively and quantitatively. For qualitative assignment, we employed two voting approaches (Ensemble-Majority and Ensemble-Unanimous) similar to those used to improve somatic variant calling [29–31]. Under Ensemble-Majority, a signature was considered present in a sample if ≥3 of 5 tools agreed that signature was active. Ensemble-Unanimous required agreement from all five tools. The latter is meant to be a stringent approach minimizing false positives. Due to its clinical relevance and heterogenous assignment across tools, we first aimed to determine if these ensemble approaches could improve qualitative SBS3 calling. Here, we utilized WGS-derived SBSs from 560 breast tumors with known *BRCA1/2* mutational status [32]. As samples with bi-allelic inactivation of either *BRCA1* (*n* = 47) or *BRCA2* (*n* = 30) are typically expected to have SBS3 activity, these samples were labeled as truth. Assigning SBS3 to patients with bi-allelic inactivation of *BRCA1/2*, Ensemble-Unanimous had a PPV that was 19.7% higher than the second-best tool (SigProfilerAssignment) as well as the highest diagnostic accuracy at ~87% (Figure 5A and C). In contrast, Ensemble-Unanimous—along with SigProfilerAssignment—had the lowest NPV likely due to its stringent definition of activity. Ensemble-Majority outperformed most tools on PPV, NPV and accuracy, and only one—SignatureToolsLib—performed better across all three metrics (Figure 5A–C). We have also assessed Ensemble-Majority and Ensemble-Unanimous on different combinations of tools via the leave-one-out approach and observed similar results (Supplementary Table 5). These results demonstrate how ensemble approaches can improve the qualitative assignment of a technically challenging [33, 34] and clinically relevant [4, 27] signature.

**Figure 6:**
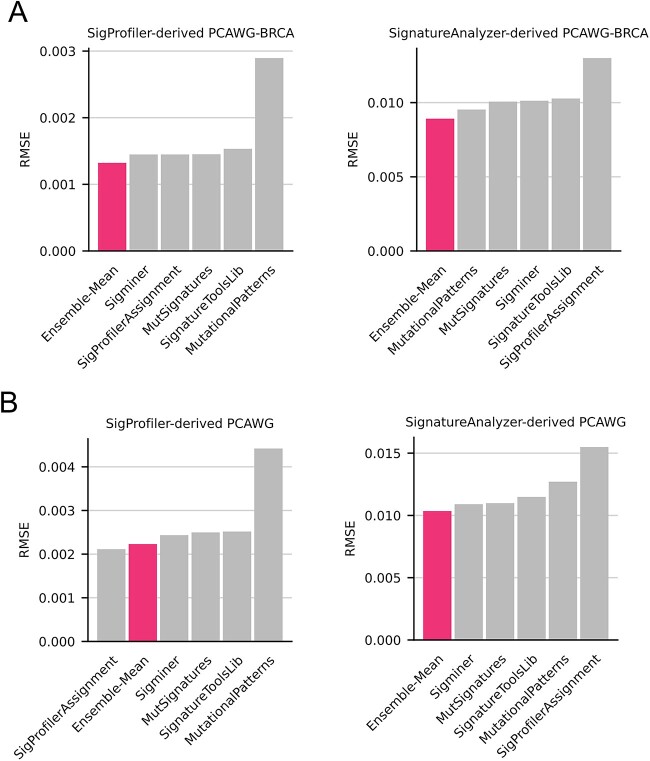
Benchmarking overall signature assignment error using synthetic datasets. (**A**) Synthetic PCAWGBRCA samples (*n* = 198), were simulated from SigProfiler’s (left) and SignatureAnalyzer’s (right) reference signature sets provided by PCAWG. Each tool was used to assign signature activities to both sets of synthetic samples. Tool performance—including Ensemble-Mean—was assessed by RMSE with lower RMSEs indicating more accurate assignment. (**B**) The same approach was repeated for pan-cancer using synthetic PCAWG samples (*n* = 2780).

We next asked if an ensemble approach could improve the quantitative estimates of all signature activities within a sample. We defined Ensemble-Mean as the bootstrap estimated mean of a signature’s activity across all tools. To test this approach, 198 mutational spectra closely mimicking signature patterns observed in PCAWG-BRCA were generated using SynSigGen (see Materials and Methods). This was done using two PCAWG signature catalogs defined by independent signature extraction tools—SigProfiler and SignatureAnalyzer. This provided two datasets of synthetic breast cancer samples with known signature activities. By comparing assigned activities to truth activities, a dataset RMSE was calculated for every tool (including Ensemble-Mean). Ensemble-Mean had the lowest assignment error for both sets of synthetic breast cancer samples (Figure 6A), demonstrating that the integration of multiple tools improves overall signature assignment accuracy. To ensure these findings are not limited to breast cancer, we generated synthetic datasets for pan-cancer samples (*n* = 2780). Ensemble-Mean had the lowest and second lowest assignment error for the SignatureAnalyzer- and SigProfiler-derived datasets, respectively (Figure 6B). Within each synthetic dataset, Ensemble-Mean provided an average assignment error reduction between 15.9% and 24.7% relative to all other tools (Supplementary Table 6). This general improvement was also seen when leave-one-out approach was applied to Ensemble-Mean (Supplementary Table 7). Notably, SigProfilerAssignment—which performed best on the SigProfiler-derived pan-cancer dataset—had the worst performance on both SignatureAnalyzer-derived datasets (Figure 6A and B). Given that SignatureAnalyzer’s SBS1 (BI_COMPOSITE_SNV_SBS1_P) and SBS5 (BI_COMPOSITE_SNV_SBS5_P) are similar but not identical to those from SigProfiler, we also conducted SignatureAnalyzer benchmarking without requiring those signatures during Refit. SigProfilerAssignment was excluded here since its underlying Refit logic, which cannot be disabled via parameterization, must include these signatures. Ensemble-mean still had the lowest and second lowest RMSE on PCAWG and PCAWG-BRCA, respectively (Supplementary Figure 9). Overall, only Ensemble-Mean had strong performance across all scenarios—suggesting that it is uniquely generalizable regardless of cancer type or reference signature catalog.

### Web portal for ensemble signature assignment

**Figure 7:**
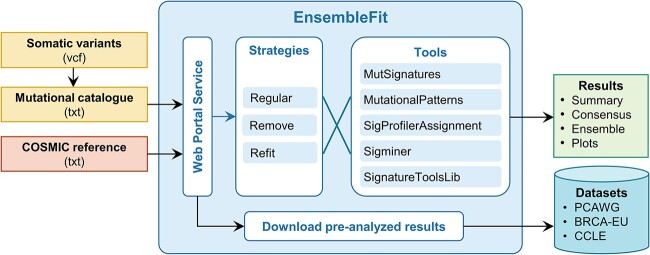
EnsembleFit web portal for consensus mutational signature assignment. Somatic variants are processed to a mutational catalog that is provided along with a mutational signature reference to the web portal service front-end. Any strategy and combination of tools can be used to generate the assignment results, which can be downloaded or analyzed within the web portal. Users can also download assignment results for valuable datasets like PCAWG, BRCA-EU and the Cancer Cell Line Encyclopedia (CCLE).

Given pervasive mutational signature assignment heterogeneity, easy-to-use platforms are required to provide comprehensive and robust results. We developed EnsembleFit (https://www.ensemblefit.pittlabgenomics.com/), a graphical web portal for ensemble-based SBS mutational signature assignment across one or more samples (Figure 7). Users can provide SBS calls as VCFs or a multi-sample mutational catalog. Both GRCh37 and GRCh38 genome builds are supported as well as multiple versions of COSMIC reference signatures. While we recommend ensemble assignment using the Refit strategy across all five tools (MutSignatures, MutationalPatterns, SigProfilerAssignment, Sigminer and SignatureToolsLib), users can employ their preferred assignment strategy over any combination of tools. EnsembleFit performs assignment through a serverless computing framework on Amazon Web Services (Supplementary Figure 10, see Supplementary Methods), which subsequently returns these results—as well as plots—to the web portal. This information can be downloaded directly or interactively explored through a reactive analytics dashboard. Consequently, users not only can obtain ensemble assignment results but also determine how their results are affected by the choice of assignment strategy and tools. EnsembleFit also provides pre-computed ensemble assignment results for PCAWG, BRCA-EU and Cancer Cell Line Encyclopedia (CCLE) (Supplementary Table 1)—enabling assignment comparisons of private data to high-value, public datasets.

## CONCLUSIONS

The lack of standardization in SBS mutational signature assignment has motivated this study to implement multiple assignment strategies across five popular signature assignment tools to assess the agreement and correlation of assignments and to benchmark the tools. Three strategies —Regular, Remove and Refit—have been popularized for SBS mutational signature assignment. Refit involves subsetting the reference signature catalog, either in a single instance or an iterative manner, to exclude signatures with low activities in a given sample. Some groups have proposed to construct this subset using a priori knowledge of tumor-specific activities [14, 17]. However, this approach is limited by the current knowledge of signature etiologies and activities, which is not ideal for samples with unique characteristics, tumors of unknown origin or understudied cancer types. We have shown that the Refit strategy excels over the others as it reduces overfitting, discards a minimal number of mutations and increases overall assignment accuracy. This assertion holds even when applied to tools lacking a native Refit implementation. Although some tools recommended the Remove strategy, our comprehensive benchmarking supports promoting Refit as the current *de facto* standard. Future work is necessary to characterize and improve upon any limitations and liabilities of Refit—especially when applied to signatures derived from other data types (e.g. indels or structural variants).

Despite uniformly applying the Refit strategy, we found that signature assignments on PCAWG-pan-cancer varied starkly across tools. This heterogeneity—which was observed both qualitatively and quantitatively—is non-negligible as it can alter biological conclusions for individual samples or cohorts. Substantial inter-tool disagreement in somatic mutation calling has been previously reported [35], and it was demonstrated that the complex interdependencies of components within whole-genome sequencing pipelines can affect cross-study reproducibility [36]. Since somatic mutation calls are a precursor to mutational signatures, errors propagating through the pipeline could compound—further disrupting confidence in interpretations. As mutational signature assignment techniques continue to be refined, it will be critical for the community to better elucidate the sources of these discrepancies.

We have demonstrated that assignment consistency is not uniform across all signatures. There have been many reported challenges in the accurate assignment of specific flat signatures such as SBS5 and SBS8 [1, 11, 18]. Similarly, we observed that flatter signatures (i.e. those with higher SDI) generally have greater assignment heterogeneity across tools. This is a crucial issue as many flat signatures have etiologies attributed to DNA damage and repair processes. One of these, the clinically relevant SBS3, is canonically used as a marker of HRD [4, 27, 28]. Tumors exhibiting a *bona fide* HRD phenotype are targetable by PARP1 inhibitors (PARPi) [37–40]. Previous studies have acknowledged co-assignment or misattribution of SBS40 with SBS3 [1, 41] and even substantially disparate attribution of SBS3 between versions 2 and 3 of the COSMIC reference signature catalog [34]. SBS3 can be supplemented by other genomic features—such as structural variants and loss-of-heterozygosity—to more accurately predict *BRCA1/2* loss and associated HRD [4, 28, 42]. Nonetheless, independent, accurate estimation of SBS3 activity is still imperative, particularly for targeted sequencing where complementary data from large structural changes are not available.

Since SBS signature assignments often show an inadequate correlation among tools, it is conceivable that each tool may perform better under different scenarios. However, as the best performing tool cannot be known a priori, we hypothesized that integration of all tools may provide more generalizable and accurate assignments. This is akin to consensus approaches that are standard practice in somatic variant calling [30, 43]. Our benchmarks have shown that the ensemble-based assignment improved qualitative assignment of SBS3 and quantitative assignment of all signatures in tumor-specific (PCAWG-BRCA) or pan-cancer (PCAWG) using both SigProfiler (COSMIC) and SignatureAnalyzer reference signature catalogs. This demonstrates that an integrative approach improves signature assignment regardless of the specific signature, dataset and reference signature catalog. It is possible that using more assignment tools could improve our ensemble approach. While we do not plan to integrate additional tools into EnsembleFit, we have open-sourced this workflow to enable others to extend our work. Whether this ensemble approach further generalizes to other systems like experimental models (e.g. cell lines and mice) or non-WGS data (e.g. exome and targeted panel sequencing) needs to be explored.

Overall, our work highlights pervasive mutational signature assignment heterogeneity across assignment strategies and tools. We demonstrate that this heterogeneity disproportionately affects critical signatures of known etiology, can confound biological conclusions and may hamper clinical applications. We provide practical recommendations to minimize these issues such as performing ensemble-based signature assignment with the Refit strategy. We have provided the EnsembleFit web portal to allow users regardless of technical background to easily adopt this approach. With the continuous evolution of the mutational signature assignment field, we assert that accurate prediction of mutational signature assignment in tumors should be standardized and tackled via crowd-sourced and community-driven competitions such as the DREAM challenges [44].

**Key Points**

- Across tools, the Refit strategy consistently performed the best for SBS mutational signature assignment.
- Tools displayed large variation in qualitative and quantitative assignments even using the same assignment strategy.
- Flatter signatures such as SBS3 demonstrate greater assignment heterogeneity across tools.
- Ensemble approaches address inter-tool variation while also improving assignment accuracy.
- The EnsembleFit web portal enables users to generate or download ensemble SBS mutational signature assignments.

## Supplementary Material

EnsembleFit_Wu_et_al_supp_info_num_rev2_bbad331

## Data Availability

All real datasets used within this study are retrievable from public databases with details provided within Supplementary Information. The EnsembleFit standalone workflow and the synthetic datasets can be found at https://github.com/pittlab-genomics/EnsembleFit. The EnsembleFit web portal service is available at https://www.ensemblefit.pittlabgenomics.com.

## Author Biographies

**Wu Jialun Andy** is a PhD student at Yong Loo Lin School of Medicine and the Cancer Science Institute of Singapore, National University of Singapore, Singapore. He is jointly supervised by Drs Ashok Venkitaraman and Jason Pitt. He is currently focused on developing methods that integrate and study multi-dimensional genomics data.

**Akila Perera** is a Software and DevOps Engineer at the Cancer Science Institute of Singapore, National University of Singapore, Singapore. His work emphasizes scalable genomic data processing and AI-assisted data democratization.

**Linganesan Kularatnarajah** is a Software Engineer at the Cancer Science Institute of Singapore, National University of Singapore, Singapore. His works are mainly related to building accessible client portals and cost-effective cloud infrastructures in the bioinformatics field.

**Anna Korsakova** is a Postdoctoral Researcher at the Cancer Science Institute of Singapore, National University of Singapore, Singapore. She completed her PhD in Biophysics and Computational Biology at Nanyang Technological University of Singapore, and she is currently focused on applications of machine learning in genomics.

**Jason James Pitt** is a Senior Research Scientist at the Cancer Science Institute of Singapore, National University of Singapore, Singapore. His primary research interest is genomic data science—particularly the development of methods to delineate the causes and consequences of genome instability in cancer.
