# Supplementary material for: Mutational signature assignment heterogeneity is widespread and can be addressed by ensemble approaches
Source: Brief Bioinform. 2023 Sep 22;24(6):bbad331. doi: 10.1093/bib/bbad331 (PMC10518036; doi:10.1093/bib/bbad331)
Supplement: EnsembleFit_Wu_et_al_supp_info_num_rev2_bbad331 [file ensemblefit_wu_et_al_supp_info_num_rev2_bbad331.docx]

**Supplementary Information**

**Supplementary Figures**

1. Overfitting of mutational signatures across Regular, Remove, and Refit strategies.
2. Non-random distribution of mutation types removed by the Remove strategy.
3. Relationship between RvO similarity and the proportion of mutations removed/unassigned.
4. Sample-wise concordance of assignments in selected individual tumor types.
5. Ranking of assignment agreement across all pairs of tools on PCAWG samples.
6. Distribution of sample-wise correlations of PCAWG samples for each pairwise tool comparison.
7. Signature-wise correlations of pan-cancer samples and their relationship to signature flatness.
8. Heatmap depicting the sample-wise correlation of APOBEC signature assignment across tools.
9. Benchmarking over signature assignment error using SignatureAnalyzer-derived synthetic datasets on a modified Refit strategy which does not force SBS1 and SBS5 into the reference subset.
10. Schematic representation of EnsembleFit architecture on Amazon Web Services (AWS).

**Supplementary Tables**

1. Datasets used in this study.
2. Assignment metrics for each tool-strategy on PCAWG (n = 2780).
3. Kolmogorov-Smirnov test for the number of signatures assigned to each sample in PCAWG (n = 2780).
4. Signature-wise correlations (Kendall Tau-b) of 21 signatures on PCAWG-BRCA samples (n = 198).
5. Benchmarking SBS3 assignment on *BRCA1/2* bi-allelic loss status in BRCA-EU (n = 560) including combinations of leave-one-out ensemble approach.
6. Benchmarking overall signature assignment error using four synthetic datasets: synthetic PCAWG-BRCA (n = 198) and PCAWG (n = 2780) using both SigProfiler-derived and SignatureAnalyzer-derived reference signature sets.
7. Leave-one-out benchmarking of overall signature assignment error using PCAWG (n = 2780) synthetic datasets on SigProfiler-derived and SignatureAnalyzer-derived reference signature sets.

**Supplementary Methods**

- Tools' assignment algorithms
- Implementation of assignment strategies for all tools
- Methods and arguments used to run the native methods of individual mutational signature assignment tools.
- Choice of signature reference set versions
- Bootstrap resampling procedures for Ensemble-Mean
- EnsembleFit architecture

Supplementary Figures


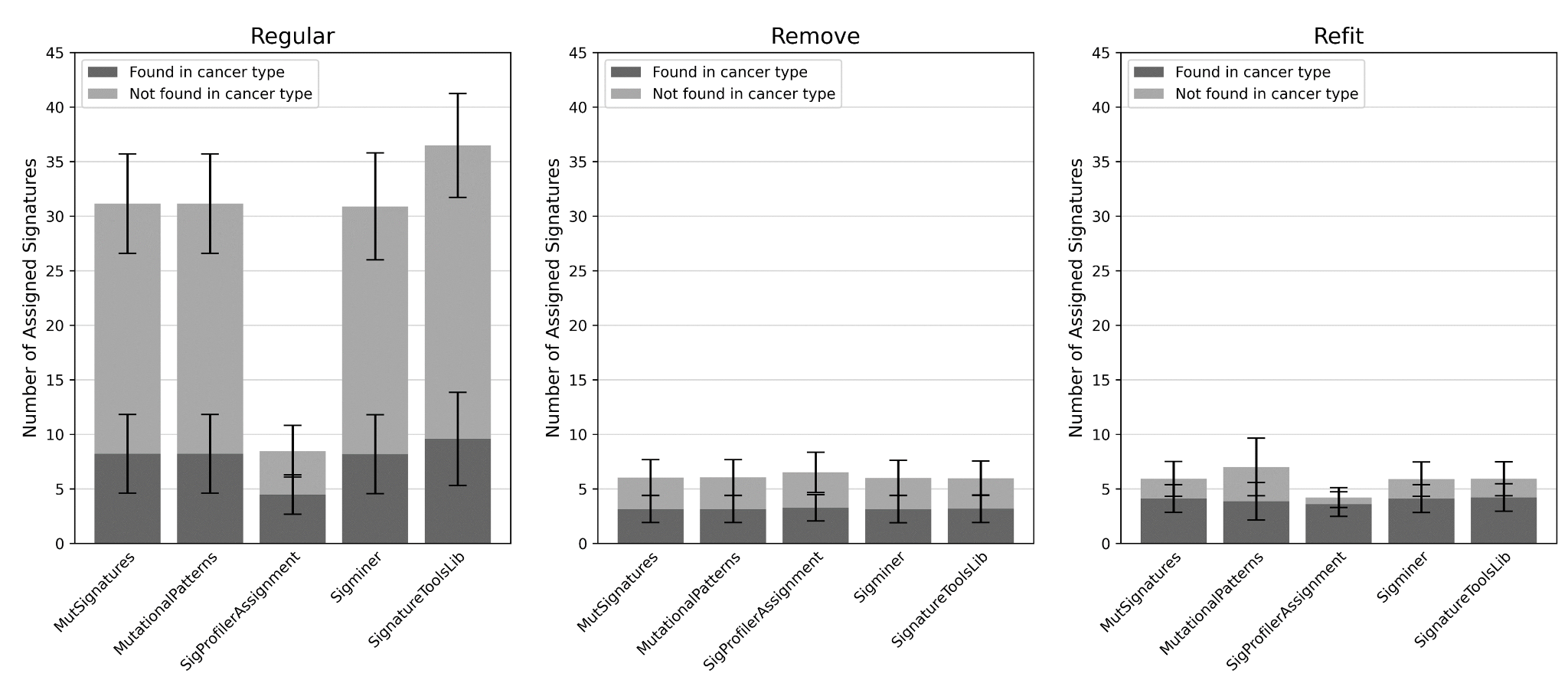


**Supplementary Figure 1. Overfitting of mutational signatures across Regular, Remove, and Refit strategies.** Aggregated mean (n = 2780) number of assigned signatures found or not found in the corresponding cancer type of the PCAWG patients, assigned by various tools (MutSignatures, MutationalPatterns, SigProfilerAssignment, Sigminer, SignatureToolsLib), and strategies (Regular, Remove, Refit). Error bar indicates the standard error of the mean (SEM).


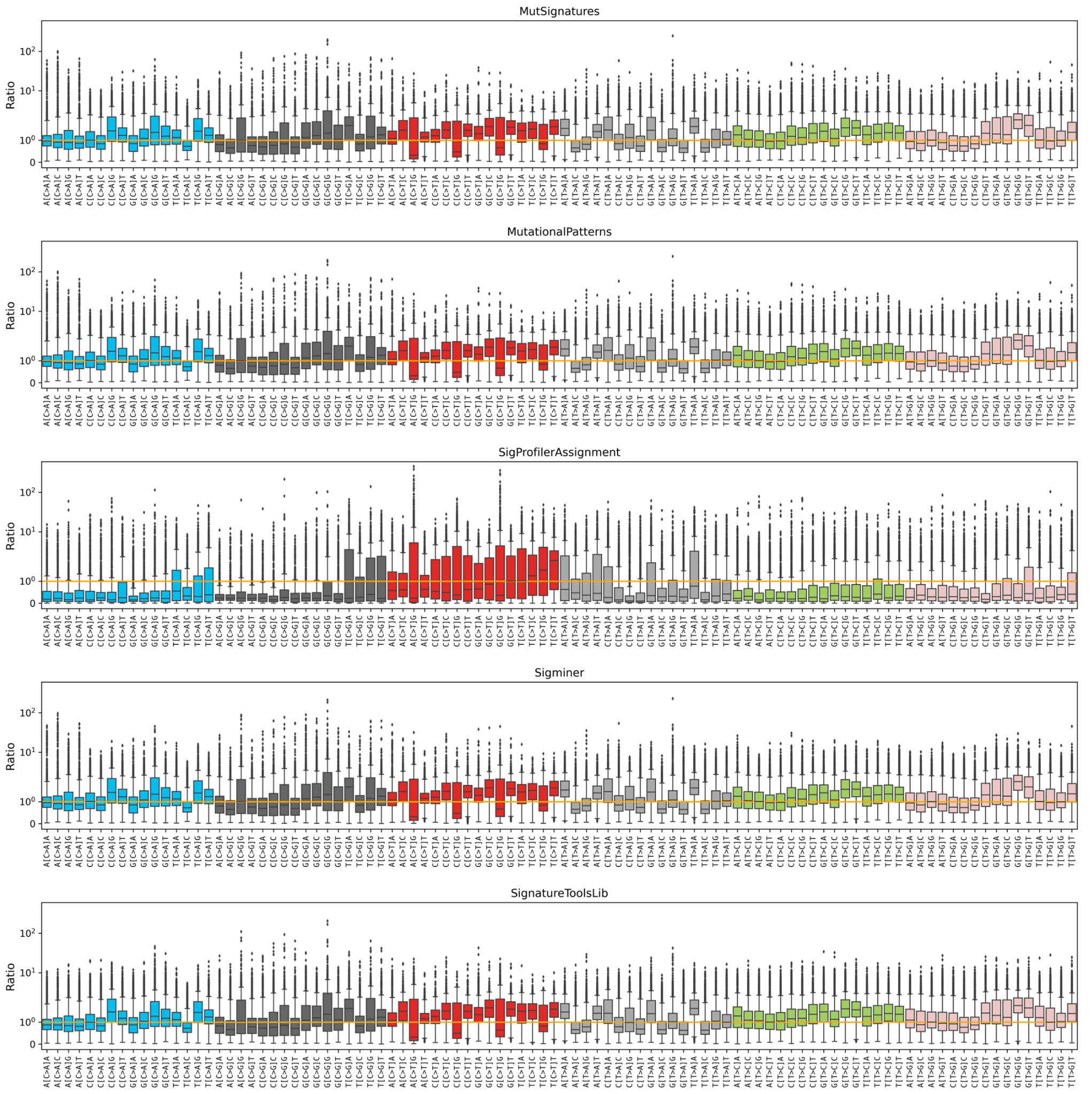


**Supplementary Figure 2.** **Non-random distribution of mutation types removed by the Remove strategy.** Across all PCAWG samples (n = 2780), the mutational profiles (96 channels of base substitutions under trinucleotide contexts) of signatures removed were recorded as a ratio to the mutational profiles of the samples. A ratio of 1, represented by the yellow line, indicates the expected contribution of the mutation types if the signature removal is random. Boxes were colored by base substitution types (C>A, C>G, C>T, T>A, T>C, T>G).


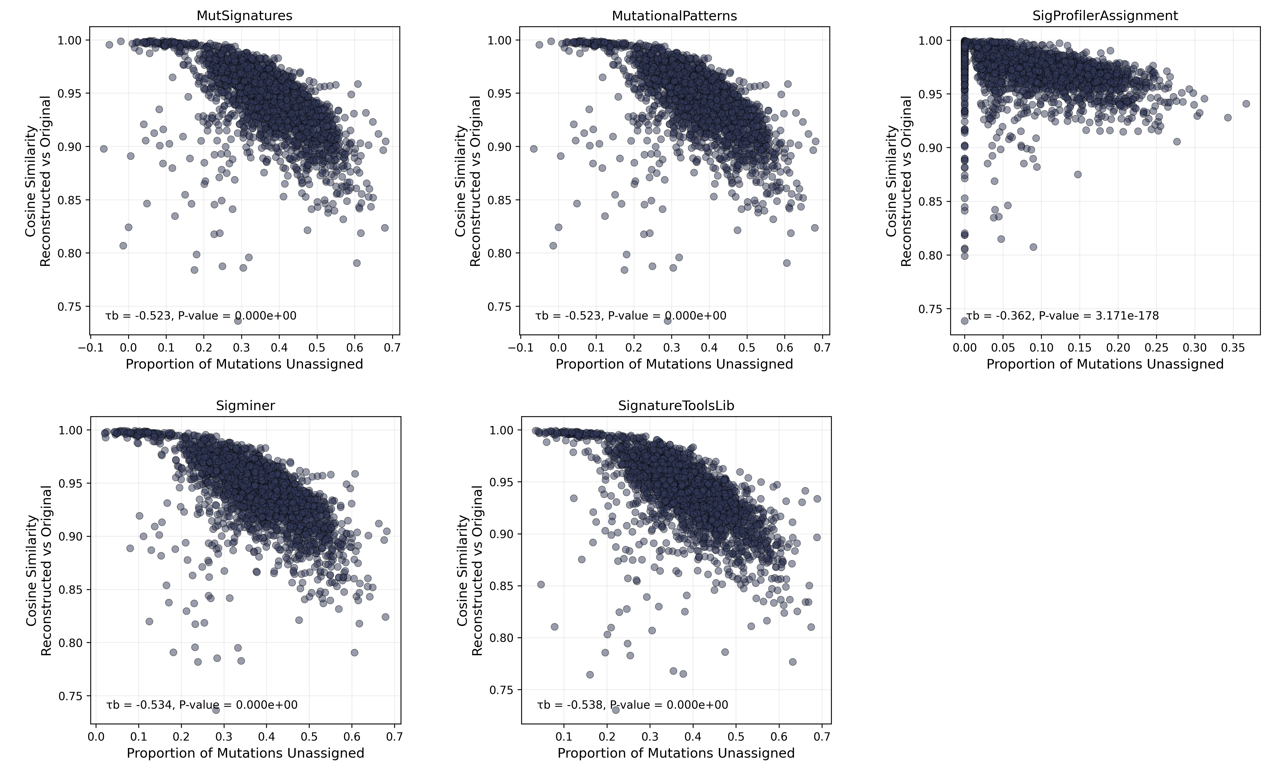


**Supplementary Figure 3.** **Relationship between RvO similarity and the proportion of mutations removed/unassigned**. Kendall Tau-B correlation and the associated P-value are reported for each tool.


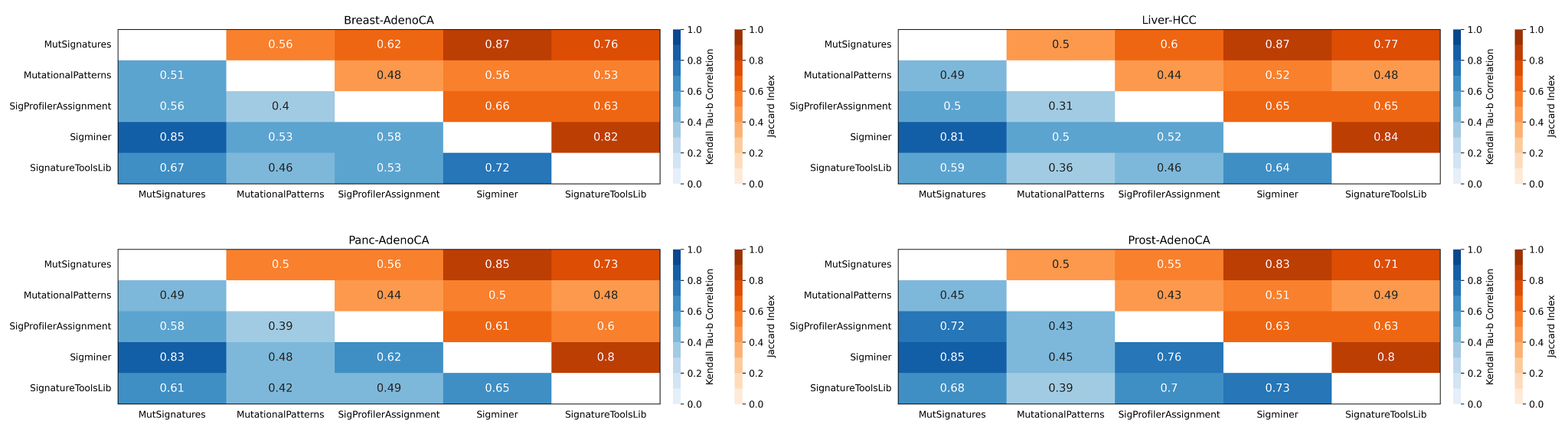


**Supplementary Figure 4.** **Sample-wise concordance of assignments in selected individual tumor types**. This includes breast adenocarcinoma (n = 198), liver hepatocellular carcinoma (n = 326), prostate adenocarcinoma (n = 286), and pancreatic adenocarcinoma (n = 241) from the PCAWG dataset. The upper right of each heatmap indicates agreement (Jaccard Index) of qualitative assignments while the lower left indicates correlation (Kendall Tau-b) of quantitative assignments.


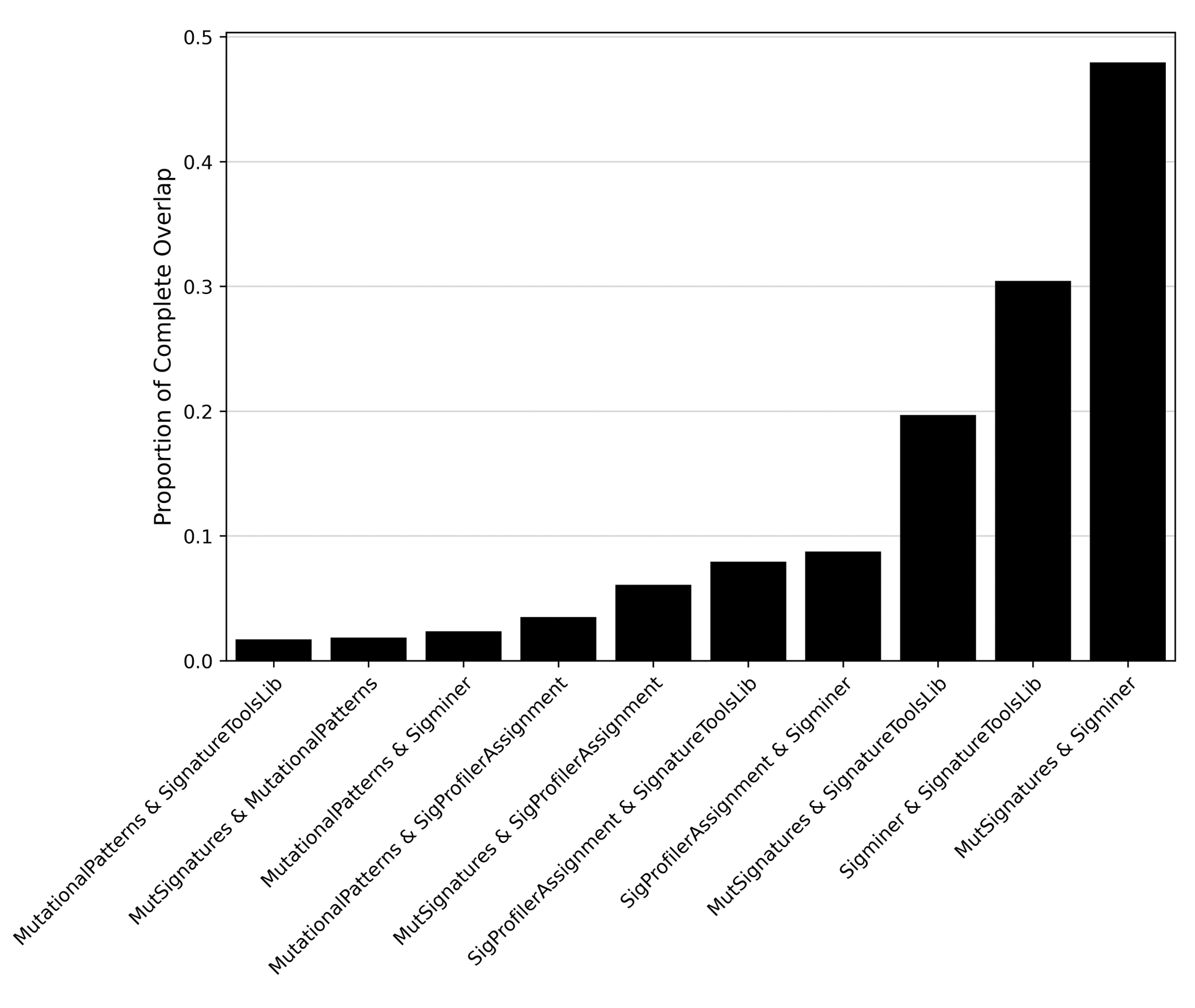


**Supplementary Figure 5.** **Ranking of assignment agreement across all pairs of tools.** The proportion of samples in PCAWG (n = 2780) where both tools in a pair have a complete overlap of assigned signatures.


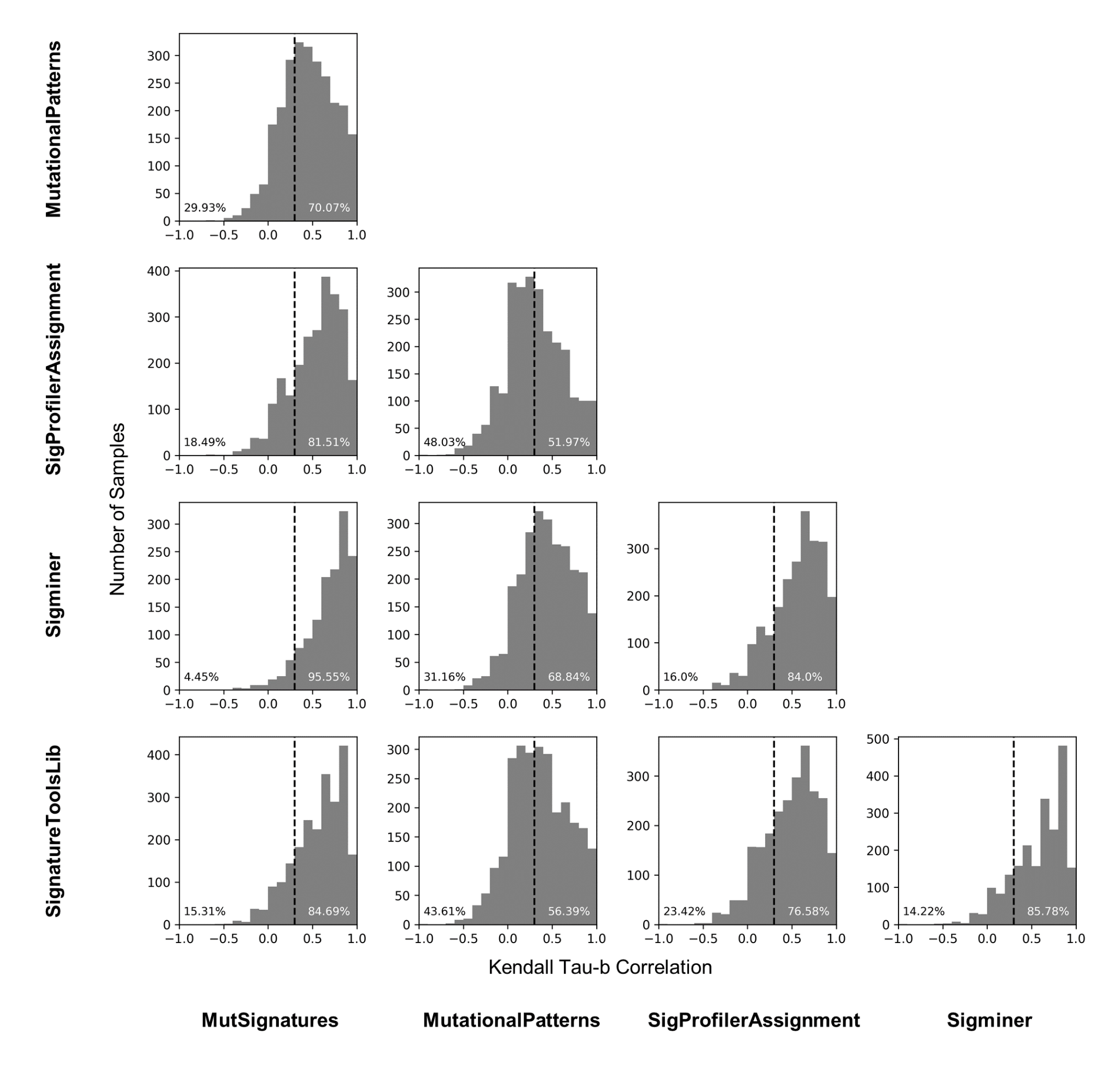


**Supplementary Figure 6**. **Distribution of sample-wise correlations of PCAWG samples for each pairwise tool comparison.** The Kendall Tau-b value of 0.3, indicated by the dotted line, demarcates the threshold for a strong positive correlation. Samples on the right are strongly and positively correlated in their signature activities assigned by the respective two tools. Samples on the left are either weakly correlated or strongly and negatively correlated. Percentage on both ends of each plot represents the percentage of the PCAWG samples (n = 2780) within each category.


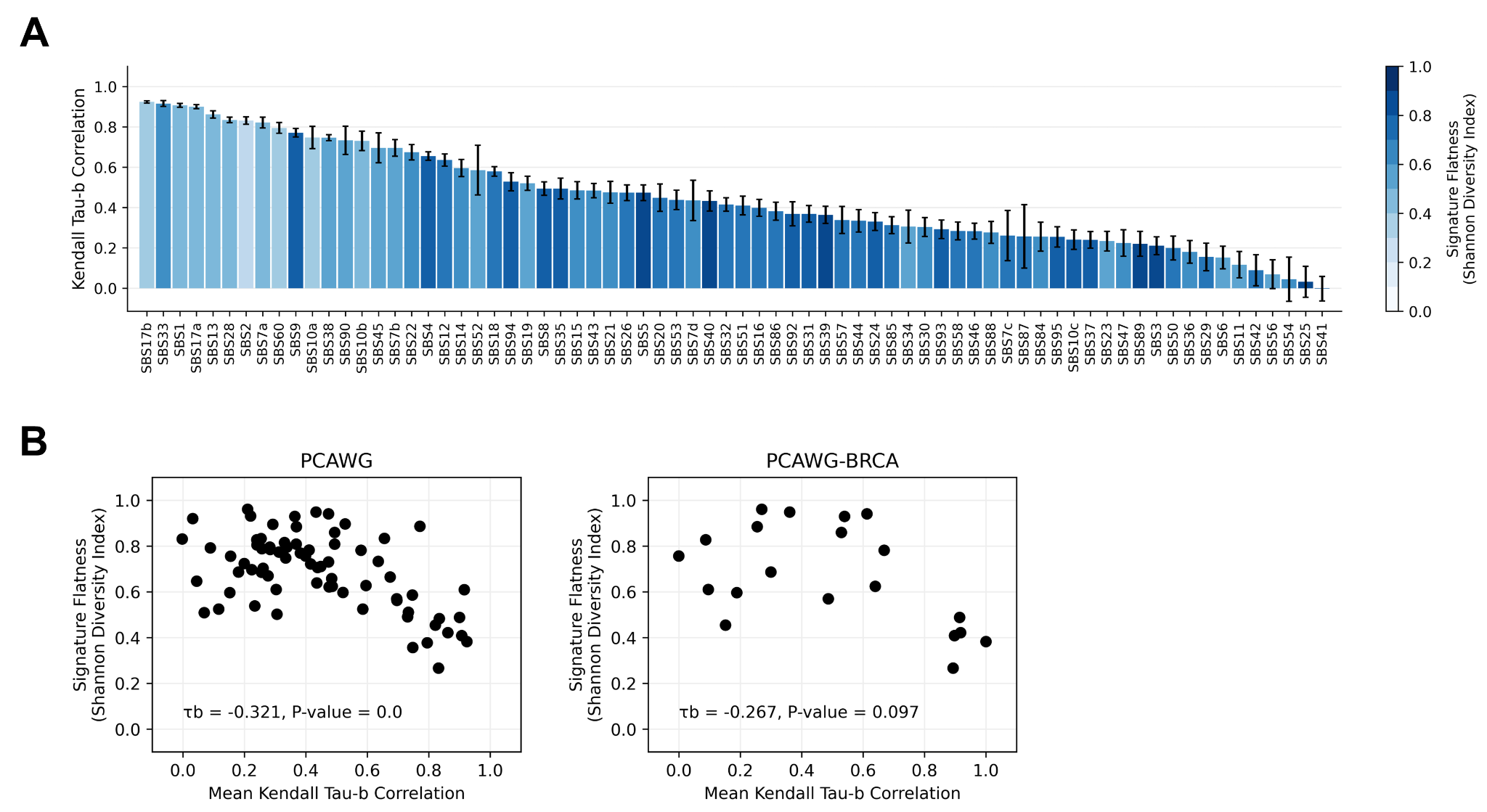


**Supplementary Figure 7. Signature-wise correlations of pan-cancer samples and their relationship to signature flatness.** (A) Signature-wise correlation (Kendall Tau-b) of assigned frequencies across all five tools in PCAWG (n = 2780). (B) Scatter plot of signatures assigned across the five tools in (left) PCAWG samples (74 signatures) and (right) PCAWG-BRCA samples (21 signatures). The signature's mean correlation (Kendall Tau-b) of assigned activities is plotted relative to the flatness of the signature profile (Shannon’s Diversity Index).


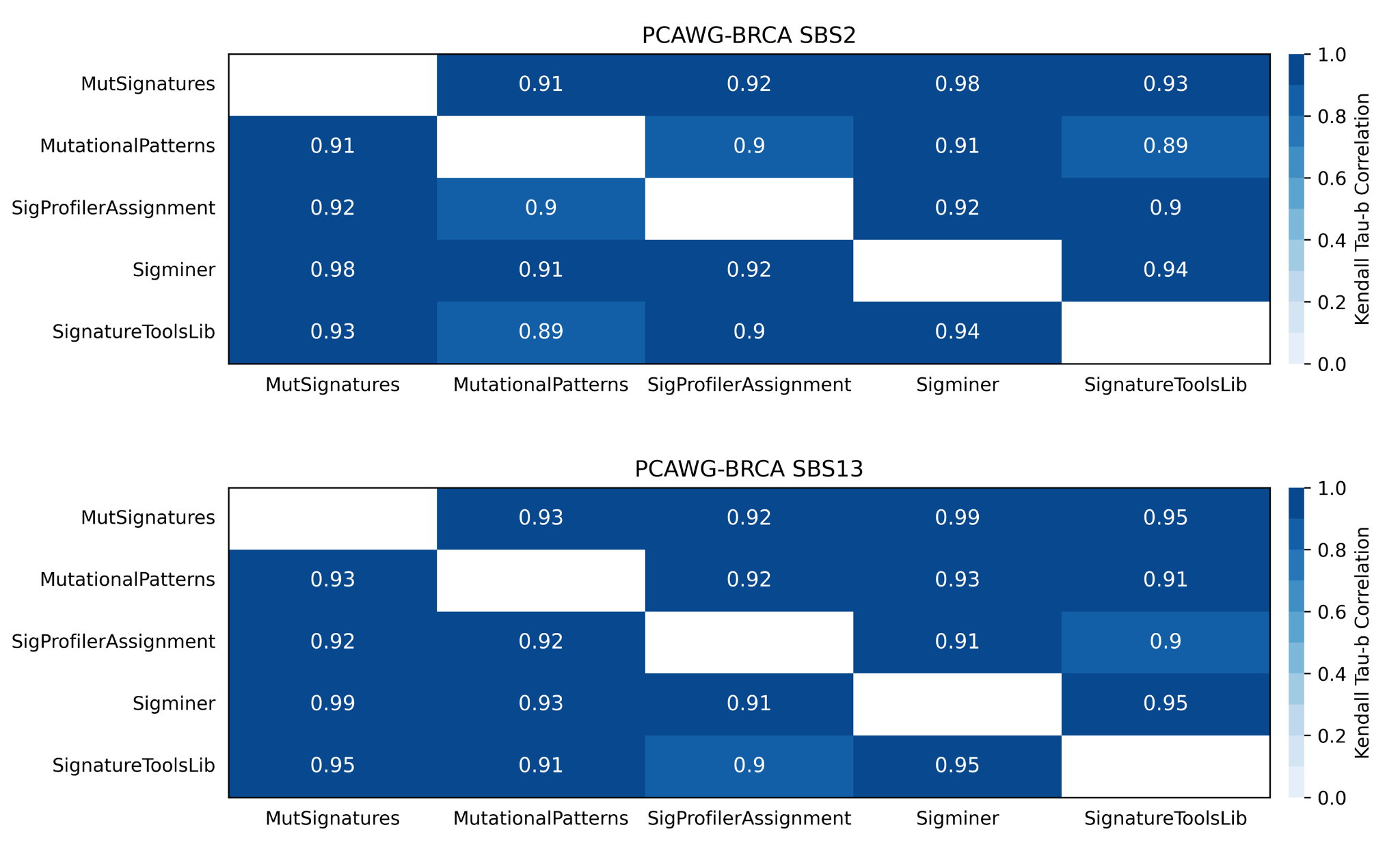


**Supplementary Figure 8.** **Heatmap depicting the sample-wise correlation of APOBEC signature assignment across tools.** SBS2 (top) and SBS13 (bottom) activity assignment across tools via mean per-sample correlation (Kendall Tau-b) in PCAWG-BRCA samples (n = 198).


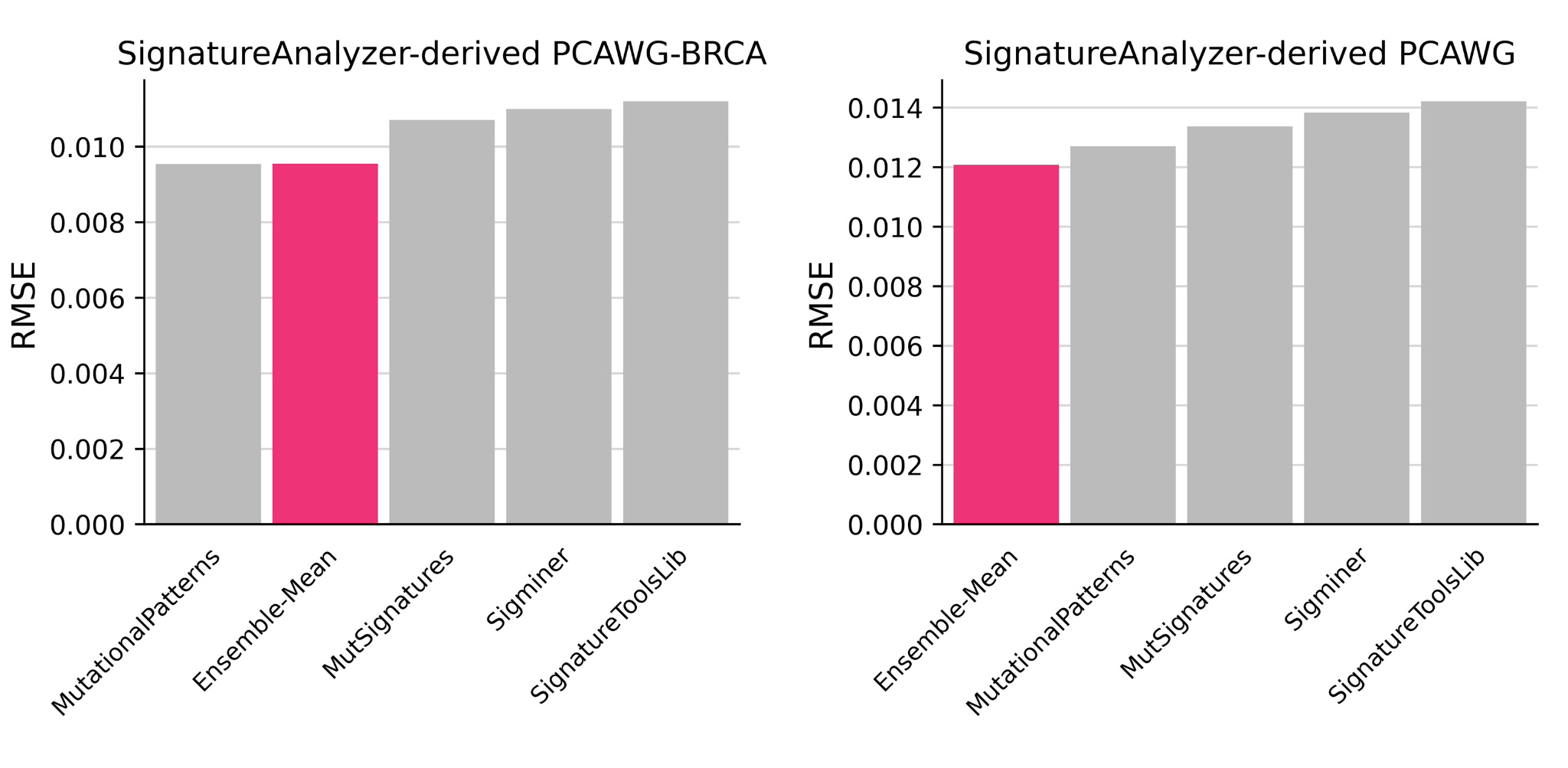


**Supplementary Figure 9. Benchmarking over signature assignment error using SignatureAnalyzer-derived synthetic datasets on a modified Refit strategy which does not force SBS1 and SBS5 into the reference subset.** SigProfilerAssignment was not included since the forced assignment of SBS1 and SBS5 cannot be disabled without modifying the source code.

[
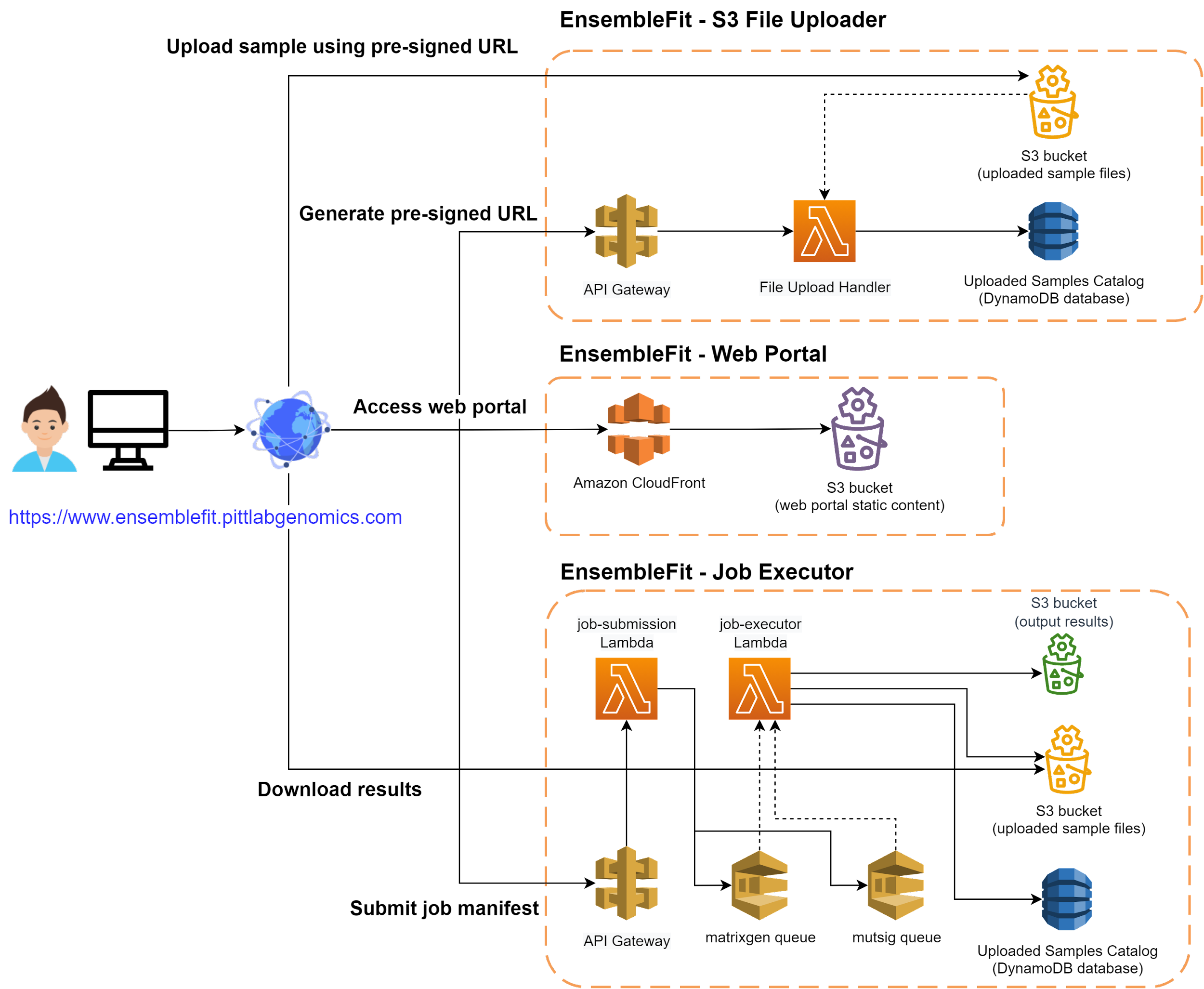
](https://app.diagrams.net/?page-id=_gZqj3NEKXgxk-RTCZ89&scale=auto#G1eDsOgy_Q8xKFAxD5TVqDoYo_AsgDhEZN)

**Supplementary Figure 10**. **Schematic representation of EnsembleFit architecture on Amazon Web Services (AWS).** EnsembleFit is a web-based software application that enables users to upload and evaluate cancer samples in either VCF or txt format. Users access the web portal at <https://www.ensemblefit.pittlabgenomics.com/> and select the sample files and profiling tools for analysis. The web portal utilizes the s3-uploader backend service to retrieve an S3 presigned URL, which is used to upload the sample files to an S3 bucket. The web application generates a configuration file based on the selected options and submits the job to the job executor backend service. The job executor downloads the corresponding sample files and executes the tools using the parameters specified in the job configuration file. The output files generated from the tools are packaged as a zip file and uploaded to a separate S3 bucket in AWS. Finally, a visual representation of the output is generated and displayed on the web portal.

Supplementary Tables

**Supplementary Table 1.** Datasets used in this study.

| **Dataset name** | **Samples** | **Download link** | **DOI** | **Remarks** |
| --- | --- | --- | --- | --- |
| PCAWG | 2780 | https://dcc.icgc.org/api/v1/download?fn=/PCAWG/mutational_signatures/Input_Data_PCAWG7_23K_Spectra_DB/Mutation_Catalogs_--_Spectra_of_Individual_Tumours/WGS_PCAWG_2018_02_09.zip | https://doi.org/10.1038/s41586-020-1943-3 | PCAWG 2020 mutational catalogue published by Working Group 7 |
| PCAWG-BRCA | 198 | https://dcc.icgc.org/api/v1/download?fn=/PCAWG/mutational_signatures/Input_Data_PCAWG7_23K_Spectra_DB/Mutation_Catalogs_--_Spectra_of_Individual_Tumours/WGS_PCAWG_2018_02_09.zip | https://doi.org/10.1038/s41586-020-1943-3 | Breast adenocarcinoma samples from the PCAWG dataset |
| BRCA-EU | 560 | https://dcc.icgc.org/api/v1/download?fn=/current/Projects/BRCA-EU/simple_somatic_mutation.open.BRCA-EU.tsv.gz | https://doi.org/10.1038/nature17676 | European Breast Cancer simple somatic mutations processed to mutational catalogue using SigProfilerMatrixGenerator |
| SP-Syn-PCAWG | 2780 | - | - | Simulated from PCAWG exposures on SigProfiler-derived signatures. Dataset mimics identical distribution of 37 cancer types in PCAWG. |
| SA-Syn-PCAWG | 2780 | - | - | Simulated from PCAWG exposures on SignatureAnalyzer-derived signatures. Dataset mimics identical distribution of 37 cancer types in PCAWG. |
| CCLE | 1702 | https://depmap.org/portal/download/all/ | https://doi.org/10.1038/s41586-019-1186-3 | Cancer Cell Line Encyclopedia (CCLE) project by the DepMap consortium |

**Supplementary Table 2.** Assignment metrics for each tool-strategy on PCAWG (n = 2780). Root mean square error (RMSE) was calculated using the published exposure on PCAWG 2020 (Supplementary Table 1) as truth.

| **Tool** | **Strategy** | **Num. COSMIC** | **Mean Num. COSMIC per Sample** | **Mean Prop. Assigned** | **RMSE** |
| --- | --- | --- | --- | --- | --- |
| SigProfilerAssignment | Regular | 66 | 8.457553957 | 1 | 0.031340714 |
| Sigminer | Regular | 67 | 30.89460432 | 1 | 0.030197575 |
| SignatureToolsLib | Regular | 67 | 36.35143885 | 1 | 0.029806112 |
| MutationalPatterns | Regular | 67 | 31.14352518 | 1 | 0.030199608 |
| MutSignatures | Regular | 67 | 31.14352518 | 1 | 0.030200276 |
| SigProfilerAssignment | Remove | 66 | 6.516906475 | 0.907345516 | 0.030318809 |
| Sigminer | Remove | 65 | 6.010071942 | 0.625288305 | 0.027133557 |
| SignatureToolsLib | Remove | 66 | 5.975539568 | 0.619682177 | 0.026804574 |
| MutationalPatterns | Remove | 65 | 6.047482014 | 0.630515651 | 0.027225674 |
| MutSignatures | Remove | 65 | 6.047122302 | 0.630442364 | 0.027226414 |
| SigProfilerAssignment | Refit | 61 | 4.20323741 | 1 | 0.023900656 |
| Sigminer | Refit | 65 | 5.894604317 | 1 | 0.025460283 |
| SignatureToolsLib | Refit | 66 | 5.927338129 | 1 | 0.024551032 |
| MutationalPatterns | Refit | 66 | 7 | 0.985718543 | 0.030579916 |
| MutSignatures | Refit | 65 | 5.91618705 | 0.989413941 | 0.025548652 |

**Supplementary Table 3.** Kolmogorov-Smirnov test for the number of signatures assigned to each sample in PCAWG (n = 2780). P-values were adjusted using Benjamini-Hochberg (BH) correction.

| **Pair** | **P-value** | **Adjusted P-value (BH)** |
| --- | --- | --- |
| MutSignatures vs SigProfilerAssignment | 0.00E+00 | 0.00E+00 |
| MutationalPatterns vs SigProfilerAssignment | 2.93E-289 | 1.47E-288 |
| SigProfilerAssignment vs Sigminer | 1.47E-238 | 4.91E-238 |
| SigProfilerAssignment vs SignatureToolsLib | 2.43E-237 | 6.08E-237 |
| MutationalPatterns vs SignatureToolsLib | 5.96E-81 | 1.19E-80 |
| MutationalPatterns vs Sigminer | 1.43E-75 | 2.38E-75 |
| MutSignatures vs MutationalPatterns | 2.23E-30 | 3.19E-30 |
| MutSignatures vs SignatureToolsLib | 9.50E-29 | 1.19E-28 |
| MutSignatures vs Sigminer | 2.90E-26 | 3.22E-26 |
| Sigminer vs SignatureToolsLib | 8.77E-01 | 8.77E-01 |

**Supplementary Table 4.** Signature-wise correlations (Kendall Tau-b) of 21 signatures on PCAWG-BRCA samples (n = 198). The Shanon Diversity Index (SDI) represents the flatness of each signature profile. Signature etiologies are based on COSMIC v3.3.

| **Signature** | **Proposed etiology (COSMIC v3.3)** | **Correlation Mean** | **Correlation SEM** | **Shanon Diversity Index (SDI)** |
| --- | --- | --- | --- | --- |
| SBS17b | Unknown | 1 | 0 | 0.382712631 |
| SBS13 | APOBEC activity | 0.917141664 | 0.011368189 | 0.421539919 |
| SBS17a | Unknown | 0.914389224 | 0.030435343 | 0.488434313 |
| SBS1 | Spontaneous deamination of 5-methylcytosine (clock-like) | 0.89715388 | 0.01542453 | 0.408805715 |
| SBS2 | APOBEC activity | 0.892695785 | 0.014398666 | 0.266948394 |
| SBS18 | Damage by ROS | 0.668387471 | 0.037795057 | 0.781493464 |
| SBS15 | MMR deficiency | 0.639206147 | 0.085508112 | 0.623749133 |
| SBS5 | Unknown (clock-like) | 0.612165814 | 0.050480829 | 0.941076703 |
| SBS39 | Unknown | 0.539555892 | 0.055972188 | 0.929555672 |
| SBS8 | Unknown | 0.529253745 | 0.053237377 | 0.859556049 |
| SBS7b | Ultraviolet light exposure | 0.486444096 | 0.115695009 | 0.56945706 |
| SBS40 | Unknown | 0.360850587 | 0.087163115 | 0.949170761 |
| SBS36 | Defective DNA base excision repair due to MUTYH mutations | 0.299083819 | 0.091277777 | 0.686715597 |
| SBS3 | Defective homologous recombination DNA damage repair | 0.269476255 | 0.073202745 | 0.96062881 |
| SBS92 | Tobacco smoking | 0.254912556 | 0.142852752 | 0.884877887 |
| SBS6 | Defective DNA mismatch repair | 0.188420247 | 0.081471659 | 0.59636569 |
| SBS7a | UV light exposure | 0.151523062 | 0.167915692 | 0.454283105 |
| SBS30 | Defective DNA base excision repair due to NTHL1 mutations | 0.09567824 | 0.113681354 | 0.610138307 |
| SBS37 | Unknown | 0.08675189 | 0.096475271 | 0.827387756 |
| SBS16 | Unknown | -0.000492363 | 0.161325315 | 0.756265098 |
| SBS32 | Azathioprine exposure | -0.263585617 | 0.166391669 | 0.721885017 |

**Supplementary Table 5.** Benchmarking SBS3 assignment on *BRCA1/2* bi-allelic loss status in BRCA-EU (n = 560) including combinations of leave-one-out ensemble approach; labeled with the suffix of "(No TOOL)" indicating the tool that was left out. MP: MutationalPatterns, MS: MutSignatures, SPA: SigProfilerAssignment, SGM: Sigminer, STL: SignatureToolsLib.

| Tool | PPV | NPV | Accuracy |
| --- | --- | --- | --- |
| Ensemble-Unanimous(No MS) | 0.55 | 0.912 | 0.873214 |
| Ensemble-Unanimous | 0.55 | 0.912 | 0.873214 |
| Ensemble-Unanimous(No SGM) | 0.55 | 0.912 | 0.873214 |
| Ensemble-Unanimous(No MP) | 0.540984 | 0.911824 | 0.871429 |
| Ensemble-Unanimous(No STL) | 0.507692 | 0.911111 | 0.864286 |
| Ensemble-Unanimous(No SPA) | 0.479339 | 0.95672 | 0.853571 |
| SigProfilerAssignment | 0.459459 | 0.911523 | 0.851786 |
| Ensemble-Majority  (No SGM) | 0.458015 | 0.960373 | 0.842857 |
| Ensemble-Majority  (No MS) | 0.453846 | 0.95814 | 0.841071 |
| Ensemble-Majority  (No STL) | 0.411348 | 0.954654 | 0.817857 |
| Ensemble-Majority  (No MP) | 0.410959 | 0.958937 | 0.816071 |
| SignatureToolsLib | 0.409357 | 0.982005 | 0.807143 |
| Ensemble-Majority  (No SPA) | 0.378882 | 0.9599 | 0.792857 |
| Ensemble-Majority | 0.378049 | 0.962121 | 0.791071 |
| Sigminer | 0.32967 | 0.955026 | 0.751786 |
| MutSignatures | 0.331522 | 0.957447 | 0.751786 |
| MutationalPatterns | 0.328125 | 0.961957 | 0.744643 |

**Supplementary Table 6.** Benchmarking overall signature assignment error using four synthetic datasets: synthetic PCAWG-BRCA (n = 198) and PCAWG (n = 2780) using both SigProfiler-derived and SignatureAnalyzer-derived reference signature sets.

| **Tool** | **Signature Reference** | **Cancer Type** | **RMSE** |
| --- | --- | --- | --- |
| Ensemble-Mean | SigProfiler | Breast Adenocarcinoma | 0.002238 |
| Sigminer | SigProfiler | Breast Adenocarcinoma | 0.002456 |
| SigProfilerAssignment | SigProfiler | Breast Adenocarcinoma | 0.002457 |
| MutSignatures | SigProfiler | Breast Adenocarcinoma | 0.002461 |
| SignatureToolsLib | SigProfiler | Breast Adenocarcinoma | 0.002600 |
| Average without Ensemble-Mean | SigProfiler | Breast Adenocarcinoma | 0.002977 |
| MutationalPatterns | SigProfiler | Breast Adenocarcinoma | 0.004910 |
| Ensemble-Mean | SignatureAnalyzer | Breast Adenocarcinoma | 0.009388 |
| MutationalPatterns | SignatureAnalyzer | Breast Adenocarcinoma | 0.010038 |
| MutSignatures | SignatureAnalyzer | Breast Adenocarcinoma | 0.010602 |
| Sigminer | SignatureAnalyzer | Breast Adenocarcinoma | 0.010661 |
| SignatureToolsLib | SignatureAnalyzer | Breast Adenocarcinoma | 0.010820 |
| Average without Ensemble-Mean | SignatureAnalyzer | Breast Adenocarcinoma | 0.011162 |
| SigProfilerAssignment | SignatureAnalyzer | Breast Adenocarcinoma | 0.013689 |
| SigProfilerAssignment | SigProfiler | Pan-cancer | 0.003583 |
| Ensemble-Mean | SigProfiler | Pan-cancer | 0.003670 |
| MutSignatures | SigProfiler | Pan-cancer | 0.004061 |
| Sigminer | SigProfiler | Pan-cancer | 0.004091 |
| SignatureToolsLib | SigProfiler | Pan-cancer | 0.004247 |
| Average without Ensemble-Mean | SigProfiler | Pan-cancer | 0.004674 |
| MutationalPatterns | SigProfiler | Pan-cancer | 0.007386 |
| Ensemble-Mean | SignatureAnalyzer | Pan-cancer | 0.01035 |
| Sigminer | SignatureAnalyzer | Pan-cancer | 0.01090 |
| MutSignatures | SignatureAnalyzer | Pan-cancer | 0.01098 |
| SignatureToolsLib | SignatureAnalyzer | Pan-cancer | 0.01149 |
| Average without Ensemble-Mean | SignatureAnalyzer | Pan-cancer | 0.01231 |
| MutationalPatterns | SignatureAnalyzer | Pan-cancer | 0.01271 |
| SigProfilerAssignment | SignatureAnalyzer | Pan-cancer | 0.01549 |

**Supplementary Table 7.** Leave-one-out benchmarking of overall signature assignment error using PCAWG (n = 2780) synthetic datasets on SigProfiler-derived and SignatureAnalyzer-derived reference signature sets. The leave-one-out Ensemble-Mean approaches are labeled with the suffix of "(No TOOL)" indicating the tool that was left out. MP: MutationalPatterns, MS: MutSignatures, SPA: SigProfilerAssignment, SGM: Sigminer, STL: SignatureToolsLib.

| **Tool** | **Signature Reference** | **RMSE** |
| --- | --- | --- |
| Ensemble-Mean (No MP) | SigProfiler | 0.003447 |
| SigProfilerAssignment | SigProfiler | 0.003583 |
| Ensemble-Mean | SigProfiler | 0.00367 |
| Ensemble-Mean (No STL) | SigProfiler | 0.003766 |
| Ensemble-Mean (No MS) | SigProfiler | 0.003781 |
| Ensemble-Mean (No SGM) | SigProfiler | 0.003787 |
| MutSignatures | SigProfiler | 0.004061 |
| Sigminer | SigProfiler | 0.004091 |
| Ensemble-Mean (No SPA) | SigProfiler | 0.004199 |
| SignatureToolsLib | SigProfiler | 0.004247 |
| MutationalPatterns | SigProfiler | 0.007386 |
| Ensemble-Mean (No SPA) | SignatureAnalyzer | 0.010688 |
| Ensemble-Mean | SignatureAnalyzer | 0.010863 |
| Ensemble-Mean (No MS) | SignatureAnalyzer | 0.010962 |
| Ensemble-Mean (No STL) | SignatureAnalyzer | 0.010993 |
| Ensemble-Mean (No SGM) | SignatureAnalyzer | 0.011032 |
| Sigminer | SignatureAnalyzer | 0.011475 |
| Ensemble-Mean (No MP) | SignatureAnalyzer | 0.011522 |
| MutSignatures | SignatureAnalyzer | 0.011562 |
| SignatureToolsLib | SignatureAnalyzer | 0.012092 |
| MutationalPatterns | SignatureAnalyzer | 0.013376 |
| SigProfilerAssignment | SignatureAnalyzer | 0.016266 |

Supplementary Methods

**Tools' assignment algorithms**

SigProfilerAssignment: *cosmic_fit*

Use Scipy's non-negative least squares (NNLS) to get the initial composition, then find a subset of reference signatures, on a per-sample basis, that have assignment above a threshold (defaults to 5% activity). SBS1 and SBS5 are added into the subset. Then, the add-remove functionality of SigProfilerExtractor's Module 5, which also uses NNLS, is performed to obtain a final composition.

Sigminer: *sig_fit*

Use own implementation of quadratic programming (QP) (*decompose_QP*) by default to optimize the fitting of all reference signatures to the sample.

SignatureToolsLib: *Fit*

Use non-negative linear models (NNLM) package to optimize the fitting of all reference signatures to the sample with Lee's multiplicative update rule method and the Kullback-Leibler divergence metric.

MutationalPatterns: *fit_to_signatures*

Use Pragma package's NNLS (*lsqnonneg*) method to optimize fitting of all reference signatures to the sample.

MutationalPatterns: *fit_to_signatures_strict*

First run *fit_to_signatures* on the samples to find a subset of reference signatures that have positive assignment (assigned activity greater than 0) across all samples. Then, on a per-sample basis, iteratively remove a reference signature with the least contribution to the previous assignment, re-run the assignment and compute the new reconstruction cosine similarity. The iteration stops when the change in cosine similarities (delta) between the current and previous iterations exceed a max_delta (defaults to 0.004).

MutSignatures: *resolveMutSignatures*

Use own implementation of fast-combinatorial non-negative least squares (FC-NNLS) (*custom_fcnnls*) method to optimize fitting of all reference signatures to the sample.

**Implementation of assignment strategies for all tools**

Regular

The Regular strategy is the assignment of the entire reference signature set (e.g. 78 signatures from COSMIC v3.3) into the samples with 0% threshold parameters. Methods used for fitting are SigProfilerAssignment’s *cosmic_fit*, Sigminer’s *sig_fit*, SignatureToolsLib’s *Fit*, MutationalPatterns *fit_to_signatures*, and MutSignatures’ *resolveMutSignatures*. For SigProfilerAssignment, the threshold could not be set to 0%, hence a minute percentage of 0.01% was selected instead.

Remove

The Remove strategy first assigns the entire reference set like a Regular fit, then any signature with assigned activity of less than 5% is removed or specifically re-assigned to an “unassigned” category. Doing so will result in a portion of the sample's mutations having no signatures assigned.

Refit

The Refit strategy also first assigns the entire reference set, then identifies signatures with assigned activity greater than 5%, termed "accepted signatures". The main difference between Refit and Remove is that Remove only filter for assignments of the accepted signatures while Refit subsets the reference to the accepted signatures for a second run of assignment. As a result, Refit has more overall assigned activity than Remove. However, the assigned activity of individual accepted signatures may increase or decrease in Refit relative to Remove depending on how the assignment tool's algorithm uses the reference signature (a full reference versus a subset reference). In addition, specifically for the COSMIC reference, the common signatures, SBS1 and SBS5, are always included in the reference subset. This approach is similar to SigProfilerAssignment's assumptions which were based on the observation that SBS1 and SBS5 are present in nearly all PCAWG samples. SigProfilerAssignment and MutationalPatterns implemented their own Refit strategy, *cosmic_fit* with default threshold and *fit_to_signatures_strict*, respectively. These native implementations were used in this study. In short, both tools iteratively remove signatures with the lowest activity until the difference between two iterations becomes larger than a set cutoff (i.e. 5%).

**Methods and arguments used to run the native methods of individual assignment tools.**

| **Tool** | **Strategy** | **Language** | **Import and Method** | **Arguments** |
| --- | --- | --- | --- | --- |
| SigProfilerAssignment | Regular | Python | from SigProfilerAssignment import Analyzer as Analyze  Analyze.cosmic_fit(sample_path, run_output_path, signature_database=reference_path, initial_remove_penalty=0.0001) | *sample_path*:Path to the sample catalogue (TXT) file  *run_output_path*: Path to the directory where the tool generates the output files  *signature_database*: Path to the reference signature (TXT) file  *initial_remove_penalty*: Threshold for removing signatures, cannot be zero. |
|  | Refit |  | Analyze.cosmic_fit(sample_path, run_output_path, signature_database=reference_path) |  |
| MutationalPatterns | Regular | R | library(MutationalPatterns)  fit_to_signatures(matrix, ref) | *matrix*: Matrix object of the sample catalogue  *ref*: Matrix object of the reference signature |
|  | Refit |  | fit_to_signatures_strict(matrix, ref) |  |
| Sigminer | Regular | R | library(sigminer)  sig_fit(matrix, sig=ref,  rel_threshold=0,  return_class="data.table") | *matrix*: Matrix object of the sample catalogue  *ref*: Matrix object of the reference signature  *rel_threshold*: Threshold, in proportion (0 to 1), for removing signatures  *return_class*: The R class of the output results |
|  | Remove | R | sig_fit(matrix, sig=ref, rel_threshold=0.05, return_class="data.table") |  |
| SignatureToolsLib | Regular | R | library(signature.tools.lib)  Fit(matrix, ref, threshold_percent=0) | *matrix*: Matrix object of the sample catalogue  *ref*: Matrix object of the reference signature  *threshold_percent*: Threshold in percentage for removing signatures |
| SignatureToolsLib | Remove | R | Fit(matrix, ref, threshold_percent=5) |  |
| MutSignatures | Regular | R | library(mutSignatures)  resolveMutSignatures(mutCountData=mutation_counts, signFreqData=mutation_signatures) | *mutCountData*: MutSignatures' object of the sample catalogue  *signFreqData*: MutSignatures' object of the reference signature |

**Choice of signature reference set versions**

We utilized the reference signature sets from ICGC PCAWG (COSMIC v3 and SignatureAnalyzer reference) for any analysis that required the published signature activities. This includes the assessment of assignment strategies and the benchmarking of tools with synthetic datasets. This is required for correct calculation of root mean squared error (RMSE) of the assigned activities relative to PCAWG's published activities. For analyses that do not require PCAWG's published activities — the assesment of sample-wise and signature-wise concordance on PCAWG samples and the assignment of SBS3 on BRCA-EU samples — we used the latest COSMIC v3.3 reference set.

**Bootstrap resampling procedures for Ensemble-Mean**

For a given sample, there are five quantitative activities (values), one for each tool, assigned for each signature. Within an iteration, five values were randomly sampled with replacement from the original set of five activities, then the mean of the sampled values was calculated. The process was repeated for 500 iterations and a final average was taken to obtain the bootstrap estimate mean value of the signature on the sample. Every signature assigned by at least one tool is subjected to this bootstrap estimate of mean. Finally, the bootstrapped estimated mean activities of all the signatures of the sample are standardized to sum to 1 (100%). Although this bootstrap resampling procedure is also able to estimate the variance of the signature's activities, the variance is not used in the Ensemble-Mean model. No hypothesis testing was conducted on the estimated distribution of activity means nor the variances.

**EnsembleFit architecture**

The EnsembleFit web portal is built and deployed on Amazon Web Services (AWS). The web application – Svelte JS framework – permits users to upload samples in VCF or TXT formats and choose their desired parameters (e.g., strategy, tools, reference build, etc). During submission, the web tool generates a JSON job manifest containing the AWS S3 location of uploaded samples and other selected parameters. A job-executor Lambda function retrieves the job manifest and runs within a Docker runtime containing all required software packages. Dynamic visualizations are built using the chartjs and plotlyjs libraries.
